# Differing structures and dynamics of two photolesions portray verification differences by the human XPD helicase

**DOI:** 10.1093/nar/gkad974

**Published:** 2023-11-02

**Authors:** Iwen Fu, Nicholas E Geacintov, Suse Broyde

**Affiliations:** Department of Biology, New York University, 24 Waverly Place, 6th Floor, New York, NY 10003, USA; Department of Chemistry, New York University, 100 Washington Square East, New York, NY 10003, USA; Department of Biology, New York University, 24 Waverly Place, 6th Floor, New York, NY 10003, USA

## Abstract

Ultraviolet light generates cyclobutane pyrimidine dimer (CPD) and pyrimidine 6−4 pyrimidone (6−4PP) photoproducts that cause skin malignancies if not repaired by nucleotide excision repair (NER). While the faster repair of the more distorting 6–4PPs is attributed mainly to more efficient recognition by XPC, the XPD lesion verification helicase may play a role, as it directly scans the damaged DNA strand. With extensive molecular dynamics simulations of XPD-bound single-strand DNA containing each lesion outside the entry pore of XPD, we elucidate strikingly different verification processes for these two lesions that have very different topologies. The open book-like CPD thymines are sterically blocked from pore entry and preferably entrapped by sensors that are outside the pore; however, the near-perpendicular 6−4PP thymines can enter, accompanied by a displacement of the Arch domain toward the lesion, which is thereby tightly accommodated within the pore. This trapped 6−4PP may inhibit XPD helicase activity to foster lesion verification by locking the Arch to other domains. Furthermore, the movement of the Arch domain, only in the case of 6−4PP, may trigger signaling to the XPG nuclease for subsequent lesion incision by fostering direct contact between the Arch domain and XPG, and thereby facilitating repair of 6−4PP.

## Graphical Abstract

**Graphical Abstract:**
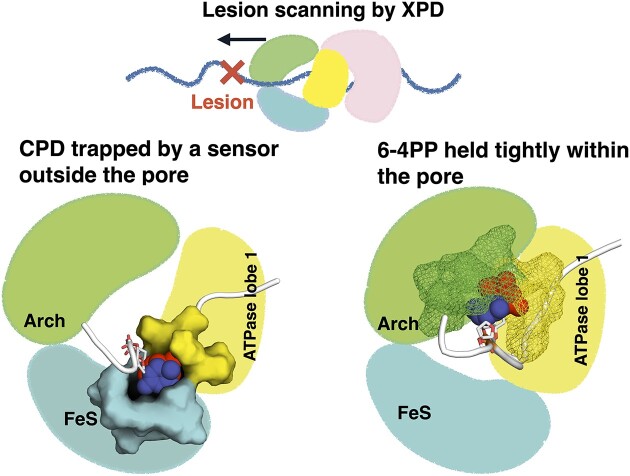

## Introduction

**Figure 1.**
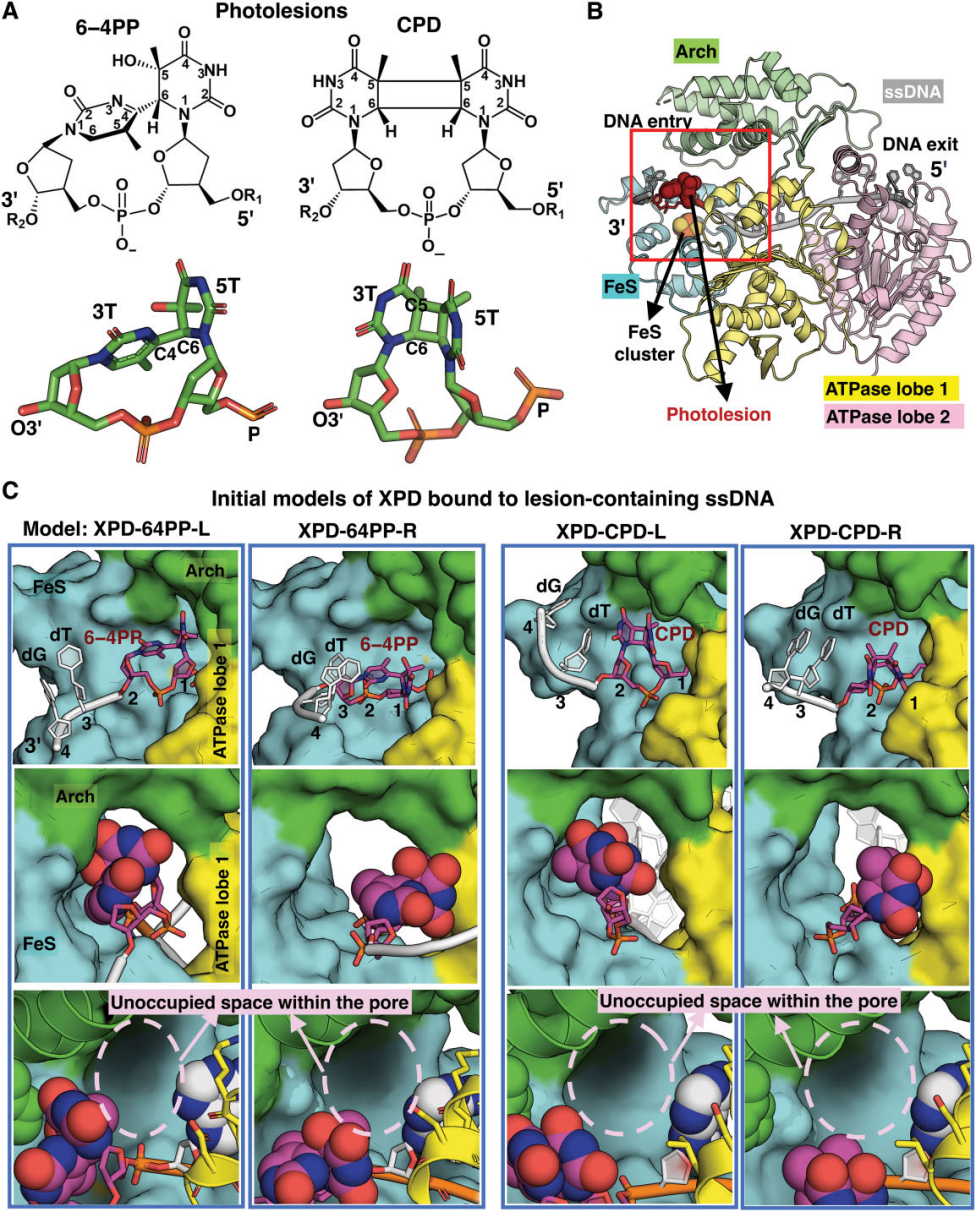
Initial structures of XPD complexed with ssDNA containing a lesion near the DNA entry pore and the positioning and orientation of the lesion. (**A**) The chemical and crystal structures of the pyrimidine (6−4) pyrimidone photoproduct (6−4PP) and the cyclobutane pyrimidine dimer (CPD). **Left**: The 6−4PP lesion, taken from a Rad4–Rad23 structure bound to 24-bp DNA containing this lesion (PDB ID 6CFI (68)), has two roughly perpendicular thymine rings, which are linked by a *single* C6−C4 covalent bond. **Right**: The CPD lesion with *cis-syn* stereochemistry, taken from the crystal structure of a nucleosome containing a CPD lesion (PDB ID 5B24 (69)), has two open book-like thymine bases that are covalently cross-linked and contains a cyclobutane ring that is formed by bonds between the C5’s and C6’s of the adjacent thymine bases. (**B**) Initial structure of XPD in complex with ssDNA (grey) containing the 6−4PP lesion (red sticks and spheres). The XPD protein contains four domains: ATPase lobe 1 (yellow), ATPase lobe 2 (pink), Arch (green) and FeS (cyan) containing an FeS cluster (indicated as orange for Fe atoms and yellow spheres for S atoms). XPD has a central tunnel that accommodates ssDNA with its 3′- and 5′-end extended toward the DNA entry and exit pores, respectively. The box highlights the DNA entry pore, formed by the Arch, FeS and ATPase lobe 1 domains. (**C**) A zoom-in view of the DNA entry pore depicts the initial structures of the XPD complex with lesion-containing ssDNA. Note that the initial structures for each model are the first snapshot after minimization in the MD simulation. Full details concerning the preparation of the initial models for the MD simulations of the XPD-ssDNA complex are given in Materials and methods.

Exposure of human skin to ultraviolet (UV) light causes damage to DNA by converting two adjacent thymines into pyrimidine dimers, generating mutagenic and carcinogenic UV-induced photolesions, the cyclobutane pyrimidine dimer (CPD) and the pyrimidine (6−4) pyrimidone (6−4PP) photoproducts (1–5) (Figure 1A), which are removed by the nucleotide excision repair (NER) mechanism (6–8). Genetic defects in NER have been associated with several autosomal recessive disorders, including xeroderma pigmentosum (XP), Cockayne (CS) and trichothiodystrophy (TTD) syndromes, which cause skin malignancies and accelerated aging (3,9–15). Patients that lack the excision repair xeroderma pigmentosum (XP) proteins that process the UV damage are extremely sensitive to UV radiation and have a several thousand-fold higher incidence of skin cancer than individuals with normal excision repair (16,17). The NER efficiencies of the photolesions differ (18–24): 6–4PP is repaired with much higher efficiency than CPD in human and other cell types (3,23–32). As a result, CPD is much more persistent and therefore the main cancer-causing lesion (33,34), while other C-containing dimers and deamination of cytosine also play contributing roles (35).

Two NER pathways eliminate the photolesions: transcription coupled NER (TC-NER) and global genomic NER (GG-NER), which differ primarily in how the lesion is initially recognized; however, they recruit the same transcription factor IIH (TFIIH) complex for lesion verification, and then converge to a common set of NER factors that form the pre-incision complex for the subsequent dual incisions, repair and ligation (6,7,15,36–38). TC-NER repairs lesions on the actively transcribed strand (12,39), in which the lesion is recognized by a stalled RNA polymerase. GG-NER repairs mainly bulky lesions throughout the entire genome. In GG-NER, the 6−4PP lesion is recognized by the xeroderma pigmentosum C protein (XPC) (7,40), while for CPD, recognition first employs the UV-damaged DNA-binding protein complex UV-DDB followed by hand-off to XPC (20,41,42). XPC that is bound to the damaged duplex recruits TFIIH, whose XPB translocase opens the DNA by translocating along the double-stranded DNA (dsDNA) in the 3′ to 5′ direction; its XPD helicase searches for the lesion by tracking along the damaged single-stranded DNA (ssDNA) in the 5′ to 3′ direction, in concert with XPA, RPA and XPG, to form a pre-incision complex (36,38,43–46). Once XPD is stalled at the lesion site, the XPF-ERCC1 nuclease is recruited and dual incision by XPF-ERCC1 and XPG takes place (47). The first cut occurs at the 5′ ds/ssDNA junction of the DNA bubble by XPF-ERCC1 and then by XPG at its 3′ junction, these cuts excise the damaged DNA segment as an oligonucleotide of 24−32 nucleotides (23,24). The restoration of the original DNA sequence by DNA polymerases/ligase using the non-damaged strand as a template completes the repair (48). Recent studies demonstrated how TFIIH reshapes its architecture that regulates XPB and XPD helicase activities depending on its functional context (e.g. transcription versus NER) (44,46) and how TFIIH cooperates with other NER factors for lesion repair (45), highlighting the role of TFIIH in the NER.

The NER susceptibilities of diverse lesions vary greatly depending on their chemical structures, stereochemistry, physical size, conformations and base sequence context (49). The CPD and 6−4PP lesions have very distinct structural features (Figure 1A): CPD features a flexible cyclobutane ring formed by two covalent bonds between the two C5 and two C6 atoms that link the two adjacent thymines, like a partly opened book; 6−4PP has a rigid covalent bond that links the C6 position of the 5′ thymine (5T) to the C4 position of the 3′ thymine (3T), with nearly perpendicular orientation of the thymine planes (50,51). Consequently, CPD imposes minimal distortion on the DNA duplex (50), while the 6–4PP produces pronounced distortion (50,52). Thus, the faster repair of 6−4PPs than CPDs in GG-NER is attributed to the more efficient recognition of 6–4PP by XPC, which is modulated by the extent of damage-induced helix destabilizing distortions (6,41,53–58). While XPC does not directly interact with the lesion during recognition since it binds the undamaged partner stand, XPD directly binds to and scans the lesion strand (36,59–64).The NER machinery must rely on the XPD-mediated verification to assure the presence of the lesion before proceeding to the excision process. Furthermore, experimental studies have suggested that differential repair of CPD and 6−4PP may depend in part on different helicase activity of XPD with these two lesions (27,65,66), to discriminate between them, which is further supported by the case of other lesions (46). Yet, whether the XPD-mediated verification of 6−4PP occurs by a different mechanism for CPD, and how these lesion-dependent differences contribute to the known faster repair of 6−4PP than CPD remain to be characterized on a dynamic, molecular level.

Hence, the focus of our work is to uncover whether XPD utilizes different strategies to verify the 6−4PP and CPD lesions and consider the possible impact on the different NER efficiencies of the two lesions. We therefore explored the conformational and dynamic possibilities of CPD and 6−4PP outside the entry pore of XPD. The entry pore, formed by the Arch domain together with the FeS and the ATPase lobe 1 domains (Figure 1B), is delineated by key residues, including the FeS residues 109−112, 128−138, 190−200, the Arch residues 373−409, and the ATPase lobe 1 residues 215−221 (Supplementary Structural Analyses). We have carried out a very comprehensive study of XPD bound to the photodamaged DNA for each lesion: 20 independent simulations, over 8 microseconds each, allowing their strict comparison under the present very extensive, identical sampling regime. With an eye to uncovering differences that are lesion-dependent, we explored the role of the lesion sensors and the Arch domain in the recognition of the damaged DNA containing each lesion. Here we found that CPD can undergo a 3′ to 5′ phosphodiester backbone translocation into the unoccupied space within the pore, but its modified bases are sterically inhibited from entering due to their quasi-stacked, open book-like topology; the CPD bases are preferably trapped by a lesion sensor near the ATPase lobe 1 and the FeS domains. For the 6−4PP lesion, we observe that its bases can translocate into the pore, accompanied by displacement of the Arch domain toward the lesion, or can be entrapped outside the pore by a lesion sensor near the Arch and the FeS domains. These extensive MD studies show for the first time that XPD responds differently to these two structurally distinct lesions. Notably, the Arch domain of XPD is more responsive when encountering the 6−4PP lesion than CPD, which may facilitate signaling for the subsequent incision step, stimulated by the direct contact mainly between the Arch domain and the XPG nuclease. Hence our work has the possible implication that differences in lesion verification by XPD may contribute to varying NER lesion susceptibilities.

## Materials and methods

### Initial models: XPD-64PP and XPD-CPD

We have previously generated a cryo-EM-based (PBD ID 6RO4 (44)) model of XPD in complex with undamaged ssDNA with an extension situated outside the entry pore of XPD (67). Importantly, this model is a translocation-capable one – it includes the incoming DNA extended outside the entry pore and it has an unoccupied space within the pore for one additional nucleotide—which permits investigation of translocation. Details concerning this model of XPD complexed with undamaged DNA are given in MATERIALS AND METHODS in our previous MD study (67).

In order to elucidate how the XPD pore responds to the CPD and 6−4PP to stall the lesion, we built two XPD models in which the ssDNA contains a lesion right outside the entry pore (Figure 1C). The 6−4PP structure is based on that in the crystal structure of a damaged DNA duplex bound to Rad4–Rad23 (PDB ID 6CFI (68)) and the CPD structure is based on the crystal structure of a nucleosome which contains CPD, with PDB ID 5B24 (69). The 5′ first two nucleotides (undamaged thymines) of the extended DNA (with sequence dTTTG) that were outside the pore in previous work (67), were replaced with a lesion with its bases 5T and 3T positioned outside the edge of the XPD pore. In both lesions, we retained the position of the 5T phosphate moiety as in PDB ID 6RO4 (39) and the modified bases outside the pore are oriented *toward* the ATPase lobe 1 helix (residues 215−221), giving a model of the XPD complexed with ssDNA containing a 6−4PP lesion (**XPD-64PP-R**) and a CPD lesion (**XPD-CPD-R**) (Figure 1C). Furthermore, to explore more conformational possibilities of the lesions outside the entry pore, we built two additional models in which the modified bases are initially oriented *away* from the ATPase lobe 1 and pointed toward the interface of the Arch and the FeS domains, yielding the models **XPD-64PP-L** and **XPD-CPD-L** (Figure 1C). These modified bases of the extended DNA outside the pore leave an unoccupied space within the pore (zoom-in view in bottom panel in Figure 1C) to permit investigation of lesion translocation and stalling in XPD.

### MD simulations

All XPD-ssDNA systems were subject to energy minimization, equilibration and ∼8 μs production MD using AMBER18 (Case *et al.*, AMBER 2018, University of California, San Francisco, 2018). To explore the conformational space of the flexible DNA extension containing the lesion, we carried out ten ∼8 μs production runs (10 × 8 μs) for each model of XPD complexed with lesion-containing DNA, resulting in a total of 20 simulations for each lesion. All MD simulations began with minimization and equilibration steps that would mitigate any strains that might be present in the initial models, ensuring the systems are equilibrated during the production step. Details concerning force field, the routine MD protocol and structural analyses are given in the Supplementary Methods and Structural Analyses. Molecular images and movies were generated with PyMOL (Schrodinger, LLC, 2015) and VMD (70). Note that in all images and movies provided, most parts of the FeS domain are displayed as transparent for the sake of clarity. All four domains (FeS, Arch and ATPase lobe 1 and 2) of human XPD helicase in PDB ID 6RO4 (44) are included in our model, except the mobile region with residues 273–325 of the Arch domain, which is not visible in the cryo-EM structure.

## Results

### Conformations and dynamics of the extended DNA containing a lesion outside the pore vary

The extended single-stranded DNA protruding outside the pore, with no coordinates deposited in the cryo-EM structure in PDB 6RO4 (44), is highly flexible with varying conformations. To comprehensively explore the conformations of the extended DNA containing a lesion in the vicinity of the entry pore, we have carried out 20 independent MD simulations of ∼8 μs each for CPD and 6–4PP. We utilized the last 2 μs to capture the properties of the equilibrated states in which both XPD and the lesion near the XPD pore achieve varying stable conformational families in the extended DNA.

#### XPD mainly retains a correctly folded pore when binding to ssDNA containing a lesion, but deviations can occur largely due to displacement of the Arch with respect to the FeS domain

During the equilibrated states, in most simulations, the XPD retains a correctly and stably folded structure with Cα RMSDs of ∼1.1–1.8 Å from its initial fold (Supplementary Figures S1 and S2). However, in 2 of 20 simulations of XPD-64PP and 1 of 20 simulations of XPD-CPD, the XPD fold shows significant deviations with RMSDs of ∼2.4–3 Å from its initial state, and the largest deviation is seen in the case of XPD-64PP. The deviations of the XPD fold mainly stem from the displacement of the Arch domain with respect to the FeS domain (Supplementary Figure S3). These results indicate that the XPD pore can be altered when encountering a lesion near the entry, while XPD always retains a correctly folded pore when binding to undamaged ssDNA (67), in line with the ability of XPD to discriminate damaged from undamaged nucleotides.

#### Extended DNA containing a lesion outside the pore is flexible

Both lesions exhibit varying deviations from their initial conformations, with heavy-atom RMSD values ranging from ∼3 to 9 Å; these deviations for both lesions stem mostly from reorientation of the modified bases, with lower 5′-P atom RMSDs of 0.8–4.8 Å (Supplementary Figures S1 and S2).

#### The 6−4PP lesion is more rigid than CPD regardless of its orientation near the entry pore

The 6−4PP is much more rigid than CPD, shown by the smaller fluctuations in the relative motions between the 3′-base (3T) and 5′-base (5T) (Supplementary Figure S4); it adopts an inflexible, propeller-like structure, with bases nearly perpendicular, due to the covalent bond between the C6 atom of the 5T and the C4 of the 3T. Due to its rigidity, its thymine-pyrimidone structure is similar to that of the initial structure in the crystal structure of the 6−4PP-containing DNA duplex bound to Rad4–Rad23 (PDB ID 6CFI (68)), reflected in their average RMSDs with values of 0.3–0.6 Å, shown in Supplementary Figure S4. This is in line with studies that showed nearly identical structures of the free (71) and the protein bound 6−4PP (72), and these are unaffected by the nature of flanking nucleotides (51). The CPD features a conformationally flexible cyclobutane ring formed by two covalent bonds between the two C5 and two C6 atoms that link the two adjacent thymines, like a partly opened, book, with thymines quasi-stacked; these thymines are more flexible than those of 6–4PP with varying deviations (RMSDs values of 0.6–1.5 Å) and greater fluctuations in relative motions between 3T and 5T when bound to XPD.

### CPD interacts with lesion sensors outside the pore entry as its bases are inhibited from entering

#### The CPD bases are mainly entrapped outside the pore by a lesion sensor near the interface between the ATPase lobe 1 and the FeS domains

In all 20 simulations, our results revealed that the bases of CPD are completely inhibited from entering the pore due to their bulky topology (Supplementary Figure S5). The CPD displays mainly three types of orientations outside the entry pore; 12 of 20 simulations (i.e. 60% of the population) showed the bases oriented toward the region at the interface between the ATPase lobe 1 and the FeS domains; in 7 of the 20 simulations (35% of the population), the modified bases are oriented toward the interface between the Arch and the FeS domains; in only one of the 20 simulations (5% of the population) are the modified bases oriented away from the FeS domain and pointed toward the gap between the Arch and the ATPase lobe 1 domains, concomitant with a narrowed gap. Thus, the steric blockage by CPD’s bulky base topology fosters its entrapment outside the pore, which supports its interaction with two nearby lesion sensors. One sensor that is most responsive to the presence of CPD is shaped by the ATPase lobe 1 residues 219−225 (DLVSKEL) and the FeS residues (R166, Y192, F193, L194, A195, R196, Y197, S198, I199, L200, H201) (Supplementary Figure S5A). When oriented in this region, the 5T base of CPD is stacked with L220 and its phosphate linkage is anchored by the Arch residue R380, thus, turning its modified bases away from the Arch domain (Supplementary Tables S1−S3). Both O2 atoms of 5T and 3T bases are hydrogen-bonded with the FeS residues R196 and/or R166. The N3 atom of the 5T base is hydrogen-bonded with the O atom carbonyl group of L220 and/or V221 (ATPase lobe 1); the O4 atom of the 5T base is hydrogen-bonded with the main chain amide of E224 and/or K223 (ATPase lobe 1). Moreover, the 5T-phosphate group of CPD is anchored stably by R196 and/or R112, preventing its backbone translocation. The second sensor is mainly shaped by the FeS residues C134, H135, T138, A139, S140, Y141, R143, Y158, Y192, F193 and the Arch residues E377, R380 and H384 (Supplementary Figure S5B). The key residues that interact with the modified bases of CPD in this region are the FeS residues H135 and S140 as well as the Arch residues R380 and H384. H135 is stacked with the 5T base of the CPD. The N atom of S140 is hydrogen-bonded to the O4 atom of the 3T base. R380 and/or H384 are hydrogen bonded with the modified bases of the CPD: the guanidinium group of R380 or the Nϵ2 atom of H384 with the O2 and N3 atoms of the 5T or 3T (Supplementary Table S3). Since these two sensors are mainly formed by the flexible side-chains of the XPD pore residues, their shapes may alter, depending upon the orientation of the lesion in the extended DNA. The third type of inhibited pore entry occurs when the modified bases of CPD are pointed away from the FeS domain and toward the gap between the Arch and the ATPase lobe 1 domains (Supplementary Figure S5C). This gap is significantly narrowed to block the bases from entering the pore, but the modified bases are not trapped by either sensor. These results suggest that the sensors may serve to initially recognize CPD, as the absence of any population manifesting translocation indicates that the barrier imposed by the sensors and the steric bulk of the lesion is unlikely to be overcome.

#### The CPD backbone can translocate in the 3′ to 5′ direction into the unoccupied space within the pore, but its modified bases are blocked from entering

**Figure 2.**
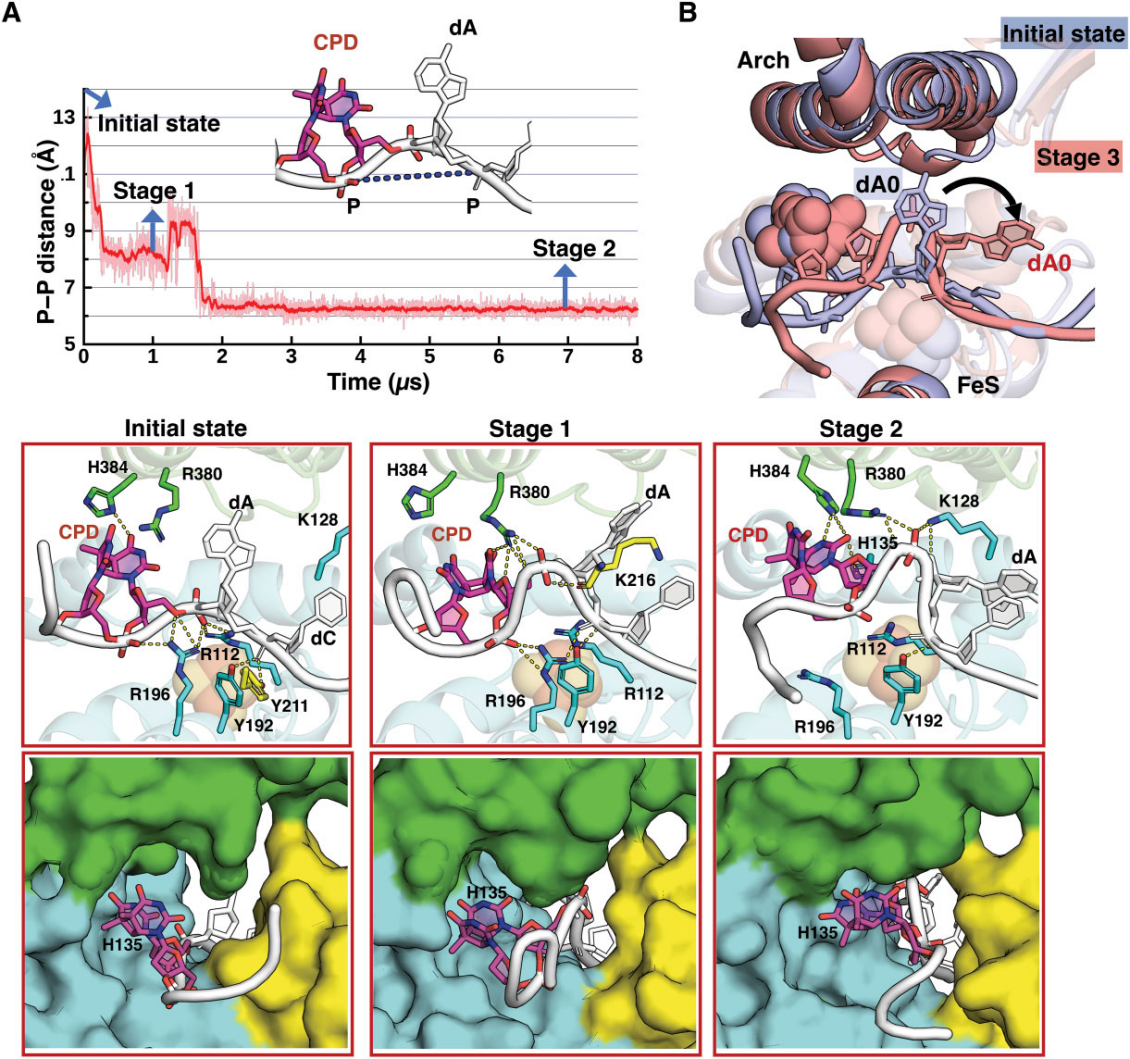
The CPD backbone is translocated in the 3′ to 5′ direction into the unoccupied space within the pore, but its modified bases are blocked from entering into the pore. (**A**) Backbone translocation of CPD occurs during 0–1.9 μs via two main DNA bending conformational transitions that take place over hundreds of nanoseconds (Supplementary MovieS1); the DNA bending is monitored by the nucleotide phosphate (crosslinked P atom of CPD) to phosphate (dA) distance. A DNA bend between the CPD and its 5′-dA occurs early in the simulation (∼200 ns). The DNA becomes more bent after 1.9 μs during the translocation and this bent DNA is then retained throughout the simulation. At the initial state (the snapshot after minimization in the MD simulations), CPD is placed outside the pore with its base pointing away from the ATPase lobe 1 domain and oriented toward the region near the Arch and the FeS domains. H384 forms hydrogen bonds with the bases of CPD. R112 and R196 anchor the backbones of CPD and dA. Y192 and Y211 interact with the backbone of dA. The nucleotide phosphate to phosphate distance is ∼ 11.6 Å. Stage 1 (during 0.2−1.9 μs): R380 and K216, both positioned on the 5′-side of CPD, together pull the 5′-phosphate group of CPD to outcompete its backbone interactions with R196 and R112. As a result, the backbone of CPD is translocated toward its 5′ direction and is lifted away from FeS and pulled toward the region near the Arch domain and ATPase lobe 1 domains. A bend is formed between dA and CPD because the phosphate group of dA is still anchored stably by Y192 after the backbone of CPD is translocated in the 3′−5′ direction. Now, R196 interacts with the crosslinked phosphate of CPD. The nucleotide phosphate to phosphate distance is ∼8 Å. Stage 2 (1.9–8 μs): K128 and R380 together pull the phosphate group of CPD to outcompete its backbone interactions with K216; as a result, the backbone of CPD is pointed away from the ATPase lobe 1 domain. The nucleotide phosphate to phosphate distance is ∼ 6.3 Å, which is retained throughout the simulation. The best representative frames from each stage are displayed, revealing the pathway of the CPD translocation in the 3′ to 5′ direction along the entry pore (Supplementary MovieS1). The top panel shows the XPD-ssDNA interactions near the entry pore; the hydrogen bonds are denoted by the yellow dashed lines. The bottom panel is a view into the entry pore (surface rendering), showing the positioning of the nucleotides near the pore during the MD simulation; the 3′-end d(TG) nucleotides are not shown for clarity. The structures are color-coded as in Figure 1. H135, R380 and H384 are labeled and displayed as sphere rendering, highlighting their relative positions with respect to CPD. (**B**) Superimposed structures with CPD prior to translocation (light-blue) and after translocation (red). A view along the entry pore showing that during the CPD transition, dA is pointed away from the position between the ATPase lobe 1 helix and the FeS helix within the pore; this creates the space for the DNA bend/loop between CPD and dA. We have superimposed the structures from stage 2 where there is a DNA bend between CPD and dA within the pore and the initial state (prior to the process of CPD translocation). The Arch domain does not lift away from the DNA; thus, it does not contribute to the extra space for the DNA bend between the CPD and dA.

In one of 20 simulations for XPD-CPD, the CPD initially positioned outside the pore undergoes a large displacement, mainly reflected in phosphodiester backbone translocation (see S1 in Supplementary Figure S1A); however, its modified bases are still blocked from entering by gating residues H135 (FeS), R380 (Arch) and H384 (Arch). The backbone translocation occurs during 0–1.9 μs via two conformational transitions that take place over hundreds of nanoseconds (Figure 2 and Supplementary MovieS1). A DNA bend between the CPD and its 5′-dA within the pore occurs early in the simulation (at ∼ 200 ns from initial state to Stage 1, Figure 2A). The DNA becomes more bent after 1.9 μs during the translocation (from Stage 1 to Stage 2, Figure 2A) and the bend is retained throughout the simulation. A superposition of the structures of the initial state and Stage 2 (Figure 2B) shows that the Arch domain is not lifted away from the DNA (which would provide extra space for the bend). Moreover, R380, H384 and H135 interact with the 5T base of the open book-like, quasi-stacked dimer, which is bulky, preventing the CPD from entering the pore.

### 6−4PP can undergo base-translocation into the DNA entry pore or interact with lesion sensors outside

#### The 6−4PP bases can translocate into the unoccupied space within the DNA entry pore to form stable interactions with the Arch domain which can manifest dynamic mobility

**Figure 3.**
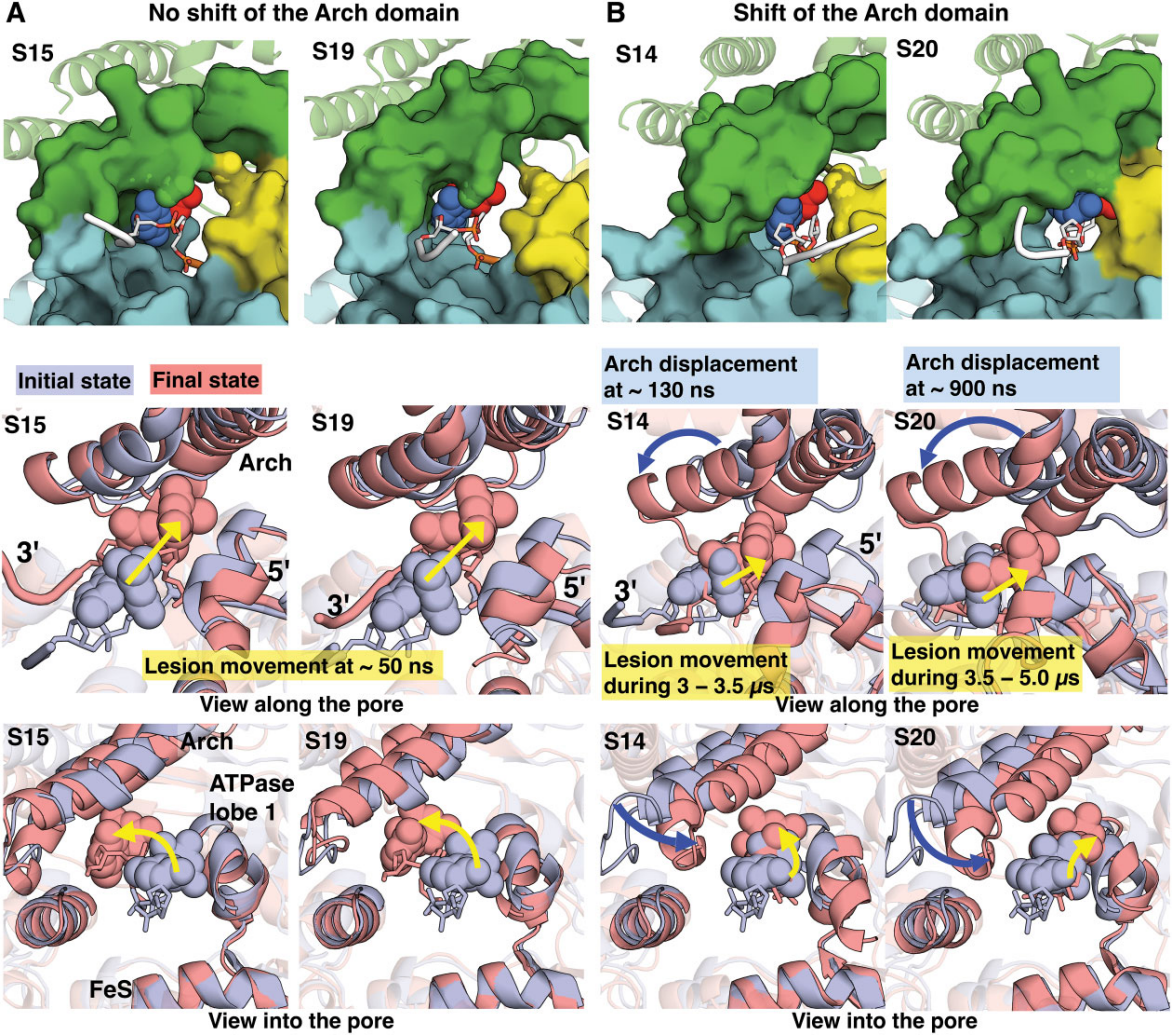
The 6−4PP lesion initially positioned outside the entry undergoes a 3′→5′ translocation via its modified bases flipping into the unoccupied space within the pore. Top panel shows the best representative structure of each simulation. Middle and bottom panels reveal the superimposed structures with 6−4PP prior to translocation (initial structure, light-blue) and after translocation (best structure, red) with a view along and into the entry pore, respectively. The movements of the Arch domain and the lesion are highlighted as dark-blue and yellow arrows, respectively. The lengths of the arrows are approximate indicators of the extents of the movements. (**A**) In simulations S15 and S19, the pathway of base-translocation of 6−4PP into the entry pore is driven by the movement of the modified bases, which takes place early in the simulation (at ∼50 ns). The bases move toward the Arch domain (bottom panel) and in a 3′ to 5′ direction (middle panel) into the pore. The Arch domain retains its initial position and shows no notable displacement during the base-translocation of the 6−4PP lesion into the pore. (**B**) In simulations S14 and S20, the pathway of base-translocation of 6−4PP involves the movements of the Arch domain (dark-blue arrow) and the lesion (yellow arrow), which take place at different times. The Arch domain is shifted toward the lesion as well as the ATPase lobe 1 and the FeS domains. The lesion is then moved in a 3′ to 5′ direction into the pore. Details concerning simulation S20 that shows the base-translocation of 6−4PP via multiple conformational transitions that take place over 5 μs is given in Figure 4. See Supplementary Figure S7 for simulation S14.

**Figure 4.**
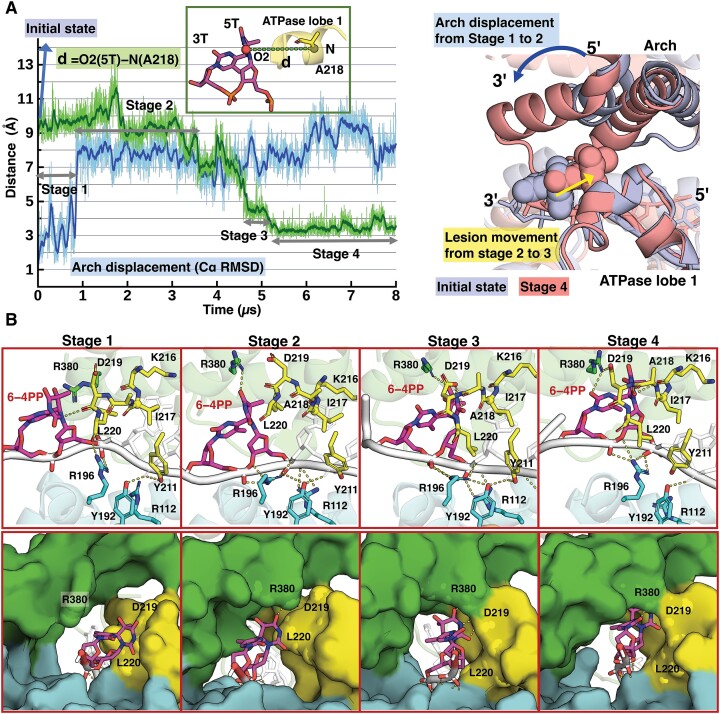
The 6−4PP is translocated in the 3′ to 5′ direction as its bases flip into the unoccupied space within the pore. **(A)** The base-translocation of 6−4PP occurs during 0–5 μs via multiple conformational transitions that take place over microseconds. Superimposed structures with 6−4PP prior to translocation (light-blue) and after translocation (red) with a view along the entry pore show that two main movements contribute to the base-translocation of 6−4PP. One is the displacement of the Arch domain (dark-blue arrow) and the other is the movement of the lesion in a 3′ to 5′ direction (yellow arrow). The plot shows the relative motion of the Arch and the FeS domains near the entry pore; this is obtained by monitoring the Cα RMSD of the Arch domain after fitting the stable region of the FeS Cα atoms to the initial structure; the Arch domain deviates significantly from its initial structure by greater than ∼8 Å at ∼0.9 μs in going from Stage 1 to Stage 2. For the movement of the lesion, we measured the distance between the base 5T O2 atom of 6−4PP and the N atom of A218 of the ATPase lobe 1 domain, **d**= O2(5T)−N(A218). The 3′→5′ movement of 6−4PP occurs at ∼3.5 μs during the transition between Stage 2 and Stage 3, which is reflected in the distance *d* decrease from ∼9 to ∼4.5 Å. **(B)** Stages 1−4 illustrate the pathway of 6−4PP’s base-translocation in the 3′ to 5′ direction along the entry pore (Supplementary MovieS2). The best representative frames from each stage are displayed. The top panel shows the XPD-ssDNA interactions near the entry pore with a view along the entry pore; the hydrogen bonds are denoted by the yellow dashed lines. The bottom panel is a view into the entry pore (surface rendering), showing the positioning and orientation of the 6−4PP lesion near the pore during the MD simulation; the 3′-end d(TG) nucleotides are not shown for clarity. The structures are color-coded as in Figure 1. R380, D219 and L220 are labeled and shown as sphere rendering, highlighting their relative positions with respect to the lesion. Stage 1 (during 0−0.9 μs): 6−4PP is placed outside the pore with its bases pointing toward the ATPase lobe 1 domain. R196 anchors the backbones of 6−4PP, and Y192 and Y211 interact with the phosphate group of 5′-dA. R380 and D219 are positioned on the 5′-side of 6−4PP. The distance of O2(5T)−N(A218) is ∼10 Å. Stage 2 (0.9–3.5 μs): after the Arch domain is shifted toward the lesion and the ATPase lobe 1 domain (Supplementary Figures S8A), R380 is hydrogen-bonded with the O4 atom of the 5T Also, R196 interacts with the 5′-P and the crosslinked P atoms of 6−4PP. Together, these interactions reorient the bases of 6−4PP away from the ATPase lobe 1 domain. The distance (*d*) of O2(5T)−N(A218) is ∼9 Å. R380 and D219 are still positioned slightly on the 5′-side of 6−4PP. Stage 3 (4.5–5 μs): 6−4PP is moved in a 3′→5′ direction closer to A218 during the transition between 3.5 and 4.5 μs, reflected in the decreased distance (*d*) of O2(5T)−N(A218) from ∼9 to ∼4.5 Å. D219 is hydrogen-bonded with N3 and O2 of 5T base. Now, both R380 and D219 are positioned on the 3′-side of 6−4PP. R196 and Y192 retain their interactions with the backbone of 6−4PP. Stage 4 (5–8 μs): the bases of 6−4PP are re-oriented toward A218, further shortening the distance *d* by ∼1 Å. Thus, the N3 and O2 atoms of the 5T base are engulfed by a small pocket formed by the ATPase lobe 1 helix with residues 215−221 (PKIADLV). R196 remains anchored to the backbone of the lesion and Y192 anchors to the backbone of 5′-dA. The extensive interactions of the XPD pore and the bases of the 6−4PP within the pore are detailed in Supplementary Figure S8B.

In 4 out 20 simulations (i.e. 20% of the population), our MD results showed that 6−4PP initially positioned outside the pore enters into the unoccupied space within the pore via translocation of its modified bases (Figure 3 and Supplementary Figure S6A and Tables S4−S6); however, its backbone is still anchored stably by the FeS residues R196 and/or R112, which inhibits the 3′→5′ backbone translocation (Supplementary Table S5). The base-translocation occurs via two different pathways that take place over different time frames (Figure 3). Figure 3A shows that the pathway of 6−4PP base-translocation into the entry pore is driven by the movement of the modified bases, which occurs early in the simulation (at ∼50 ns); the bases reorient toward the Arch domain and also move in a 3′ to 5′ direction. The Arch domain retains its initial position and shows no notable displacement during the translocation. Figure 3B displays that the pathway of base translocation involves movements of the Arch domain and the lesion; these motions take place at different times, resulting in multiple conformational transitions during this process (see S20 in Figure 4 and S14 in Supplementary Figure S7). When there is a significant displacement of the Arch domain from its initial state, the base translocation of 6−4PP occurs during 0–5 μs via multiple conformational transitions that take place over microseconds (Figure 4 and Supplementary MovieS2); the shift of the Arch domain occurs at ∼0.9 μs and the 3′→5′ movement of 6−4PP occurs over the course during the 3.5 to 5 μs interval.

When the bases of the 6−4PP lesion are translocated into the pore, they form close contacts with the Arch domain and the 5′-phosphate group is anchored stably by the FeS residues R196 and/or R192, together locking the Arch domain to the FeS domain to form a closed, less flexible pore. Notably, in one of these four simulations which revealed the base translocation into the pore, we observed a significant displacement of the Arch domain from its initial state: it is shifted toward the ATPase lobe 1 and the FeS domains as well as the 3′-DNA (Supplementary Figure S8A), resulting in a closed pore, which tightly binds the 6−4PP lesion. A detailed view of 6−4PP tightly bound by the XPD pore is presented in Supplementary Figure S8B. The modified bases are clamped tightly between the Arch and the ATPase lobe 1 domains and form two and a half hydrogen bonds with the XPD pore residues (see S20 in Supplementary Tables S5−S6). The side-chains of the Arch residues R380, H384, L391, T398, N402 and the ATPase lobe 1 residues 215−220 form stable van der Waals interactions with the bases of the 6−4PP (left panel in Supplementary Figure S8B). In the 5T base of 6−4PP, the O2 atom of 5T is hydrogen-bonded to the N atom of D219 and its N3 atom is hydrogen-bonded to the OD1 and OD2 atoms of D219; in the 3T base, the N3 and O2 atoms are hydrogen-bonded to the R380 Nη1 atoms (middle panel in Supplementary Figure S8B). Due to the nearly perpendicular, rigid bases of 6−4PP, R380 is accommodated neatly with its side-chain stacking with the 5T base and it is also hydrogen-bonded with the 3T base (right panel in Supplementary Figure S8B). Also, it's side-chain crosses over the 3T base to point toward D219 of the ATPase lobe 1 domain, resulting in a rigid and closed XPD pore. This conformation would not be feasible if replaced with CPD as its 3T base that is quasi-stacked with its 5T base would make it too bulky and sterically interfere with the orientation of R380. Furthermore, both phosphate groups of the 6−4PP lesion are anchored stably by R196 of the FeS domain (see S20 in Supplementary Table S5C). Altogether, these interactions indirectly lock the Arch domain to the FeS domain and greatly reduce the mobility of the Arch domain with respect to the FeS domain. This in-the-pore structure is in line with our previous MD study in which 6−4PP was initially situated within the pore and XPD immediately immobilized it (67); however, here our extensive, long conformational sampling allowed us to actually observe the 6–4PP bases translocating into the unoccupied space within the pore.

#### Modified bases of 6−4PP that are blocked from entering the pore are mainly entrapped by a lesion sensor near the interface between the Arch and the FeS domains

Sixteen out of 20 simulations revealed blockage of the 6–4PP lesion from entry into the unoccupied space within the pore (Supplementary Figure S6B−D and Tables S4−S6). Unlike the CPD cases, we found that if blocked, 6−4PP is mainly bound by the sensor near the Arch and the FeS domains (11/16 simulations i.e. ∼ 69% of the population) (Supplementary Figure S6B); two simulations showed the 5T base of 6−4PP bound by the region of the FeS domain (Supplementary Figure S6D), and 3 simulations revealed blockage of 6−4PP by a closed gap between the Arch and the ATPase lobe 1 domains (Supplementary Figure S6C). The sensor that preferably interacts with the 6−4PP lesion is mainly formed by the Arch residues E377, R380, S381 and H384 as well as the FeS residues C134, H135, T138, A139, S140, Y141, R143, Y158, Y192 and F193. In this sensor, the key residues that interact with the bases of 6−4PP are the FeS residues H135, T138, S140 and Y158 and the Arch residues E377, R380 and H384 (Supplementary Table S6). H135 is stacked with the 5T base of the 6−4PP. T138, S140 and Y158 or R380 and H384 are hydrogen bonded with the modified bases of the 6−4PP. And, due to the flexible side-chains of the pore residues, the shape of the sensor pocket and its interactions with the lesion may vary, depending upon the orientation of the lesion. In most cases, the modified bases of the 6−4PP are in contact with the Arch domain, further suggesting that the Arch domain plays an important role in the verification of the 6−4PP lesion. This newly discovered entrapment of 6−4PP bases by lesion sensors outside the pore adds to our prior work (67) where the ‘trapped in the pore’ structure was first uncovered, while its base-translocation pathway was first seen here (Figures 3 and 4). Thus, our current work adds the important value of multiple independent, extensive simulations that permit sampling the conformations and dynamics of the lesion as thoroughly as possible. These results, combined with those above revealing blockage of CPD outside the pore uncover differing responses of the lesion sensors to the presence of the structurally distinct lesions.

## Discussion

XPD is a key player in the NER machinery for verifying DNA lesions to avoid erroneous dual incisions on damage-free sites in NER (36,59–64). Several studies showed that when encountering a bulky lesion on the translocated strand, the helicase activities of yeast and archaeal XPD homologs as well as human XPD with intact TFIIH are inhibited at the damaged sites (59–63). The DNA entry pore, formed by the Arch domain together with the FeS and the ATPase lobe 1 domains, plays an important role in helicase activity, damage verification and NER efficiency (64,73–79). Several studies have yielded a number of significant insights on the role of the Arch dynamics in XPD’s helicase activity (67,74,80,81). Ghoneim and Spies showed that, when DNA bound, the Arch domain undergoes thermally driven open-close transitions that slightly favor the closed state with CPD present (74). Constantinescu-Aruxandei *et al.* showed that covalently crosslinking the Arch domain to the FeS domain in a closed state inactivates the helicase activity of XPD (80). A recent MD study of XPD helicase activity by Yu and coworkers demonstrated that the passage of undamaged ssDNA at the entry pore is gated by the opening and closing motions of the Arch and FeS domains during the ATP hydrolysis cycle (81). In a previous all-atom MD study we provided dynamic molecular characterizations of the XPD helicase activity with unmodified DNA (67): the Arch domain exhibits dynamic movement with respect to the FeS domain, concomitant with great pore enlargement, to allow translocation. We also found that undamaged DNA outside the pore can be temporarily inhibited from translocating via entrapment of a native base by sensors, suggesting that these sensors present a modest kinetic barrier to translocation for the unmodified DNA that may regulate its translocation rate (82). In addition, the prior work characterized one important sample of XPD helicase inhibition by the 6−4PP lesion via a ‘trapped in the pore’ structure (67). The present comprehensive work presents the first atomistic and dynamic characterization and comparison of a pair of topologically very different UV lesions as they are encountered by XPD.

In the current work, we wished to elucidate how the XPD helicase activity is differently inhibited in the presence of the CPD and 6−4PP lesions; these lesions with very different structures have strikingly different GG-NER efficiencies: 6−4PP is well repaired while the CPD that is generated in several-fold greater amount is poorly removed (3,23–32). Here we extensively explored, with the use of 20 independent simulations of ∼ 8 μs, the conformational and dynamic possibilities of each lesion near the entry pore of XPD and characterized the corresponding key XPD-lesion interactions. Remarkably, we found that the open book-like thymines of CPD are sterically blocked from the entry and preferably captured by the sensors outside the entry pore, while the nearly perpendicular thymines of 6−4PP can translocate into the entry pore and be tightly accommodated within, accompanied by a notable displacement of the Arch domain toward 6−4PP. We broadly unveiled the verification processes for these two lesions and how they are strikingly different, including how both lesions can be trapped by lesion sensors outside the entry pore, movements of the crucial XPD Arch domain, and the dynamic translocation of each lesion as it enters the entry pore. These differences support the concept that the mechanism by which XPD verifies 6−4PP is different from that CPD.

Our 20 independent and 8 us long simulations of XPD bound to each lesion-containing ssDNA revealed that the modified bases for both lesions initially positioned outside the pore are mostly blocked from translocating (revealed in 16 of 20 simulations, *i.e*. 80% of the population for 6−4PP and 100% of the population for CPD); in both cases this occurs mainly via entrapment of the modified bases by lesion sensors, thereby supporting the function of such sensors identified in other studies (59,64,73,83) and suggested by our previous MD study with undamaged DNA (67). For CPD, the sensors may serve to initially recognize this lesion, as the absence of any population manifesting translocation into the pore indicates that the barrier is significant. For 6−4PP, this sensor-governed blockage to pore entry by the 6−4PP bases was first uncovered in the present work; this emphasizes the important value of our present multiple, independent and extensive simulations that permit sampling the conformations and dynamics of the lesion outside the pore as comprehensively as possible. Furthermore, the modified bases of 6−4PP are preferably oriented toward the sensor near the region between the Arch and the FeS domains (Supplementary Figure S6B), while those of CPD are preferably oriented toward the sensor near the interface between the ATPase lobe 1 and the FeS domains (Supplementary Figure S5A), suggesting that the roles of these sensors in the recognition of damaged DNA are lesion-dependent. Notably, when CPD is bound to this preferred sensor (Supplementary Figure S5A), the recognition of its bases takes place through a number of hydrophobic and hydrogen bond interactions with the FeS residues R196 and/or R166 (FeS) as well as the ATPase lobe 1 residues L220, V221, S222, K223, E224. Moreover, the CPD phosphate linkage is anchored by R380 and the 5T-phosphate group of CPD is anchored stably by R196 and/or R112; abolishing the R196-DNA interactions leads to a one nucleotide 3′−5′ backbone-translocation into the pore as seen in one of the CPD cases (Figure 2). Overall, our MD study suggests that R196 is one of the key players in recognizing CPD right outside the entry. Our findings are consistent with biochemical findings by Mathieu *et al.* (64); they identified the key residues R196 and Y192 as delineating the lesion sensor pocket; furthermore, mutants R196E and Y192A disabled the ability of XPD to sense CPD, failed to form the expected stable complex with the lesion site and caused diminished repair of CPD.

A further striking observation in our current simulations of 6−4PP bound to XPD is that 4 of 20 simulations revealed a dynamic base-translocation process of the 6−4PP lesion into the unoccupied space within the pore (in prior work the lesion was initially positioned within the pore, which modeled the situation after translocation (67)), and the dynamic mechanism of base-translocation was first elucidated here (Figure 3 and Supplementary MovieS2). However, for CPD, base translocation was not observed, likely due to its quasi-stacked bases, which are trapped more stably by the lesion recognition sensors outside the pore than 6−4PP, as they are too bulky to enter. Furthermore, the 6−4PP lesion, that is less trapped by the sensors, is gripped tightly particularly by the Arch domain following the translocation into the pore. In particular, one simulation revealed that the Arch domain is shifted significantly toward the 6−4PP modified bases as well as the ATPase lobe 1 and the FeS domains, resulting in a narrow, rigid pore that immobilizes the 6−4PP lesion –the ‘trapped in the pore’ state (Supplementary Figure S8), which also reflects the biological importance of rare conformations (84). Such a notable movement of the Arch domain is not observed in the case of CPD, indicating that the Arch domain is more responsive to 6−4PP than to CPD and highlighting that the role of the Arch domain in the recognition of damaged DNA is lesion-dependent.

**Figure 5.**
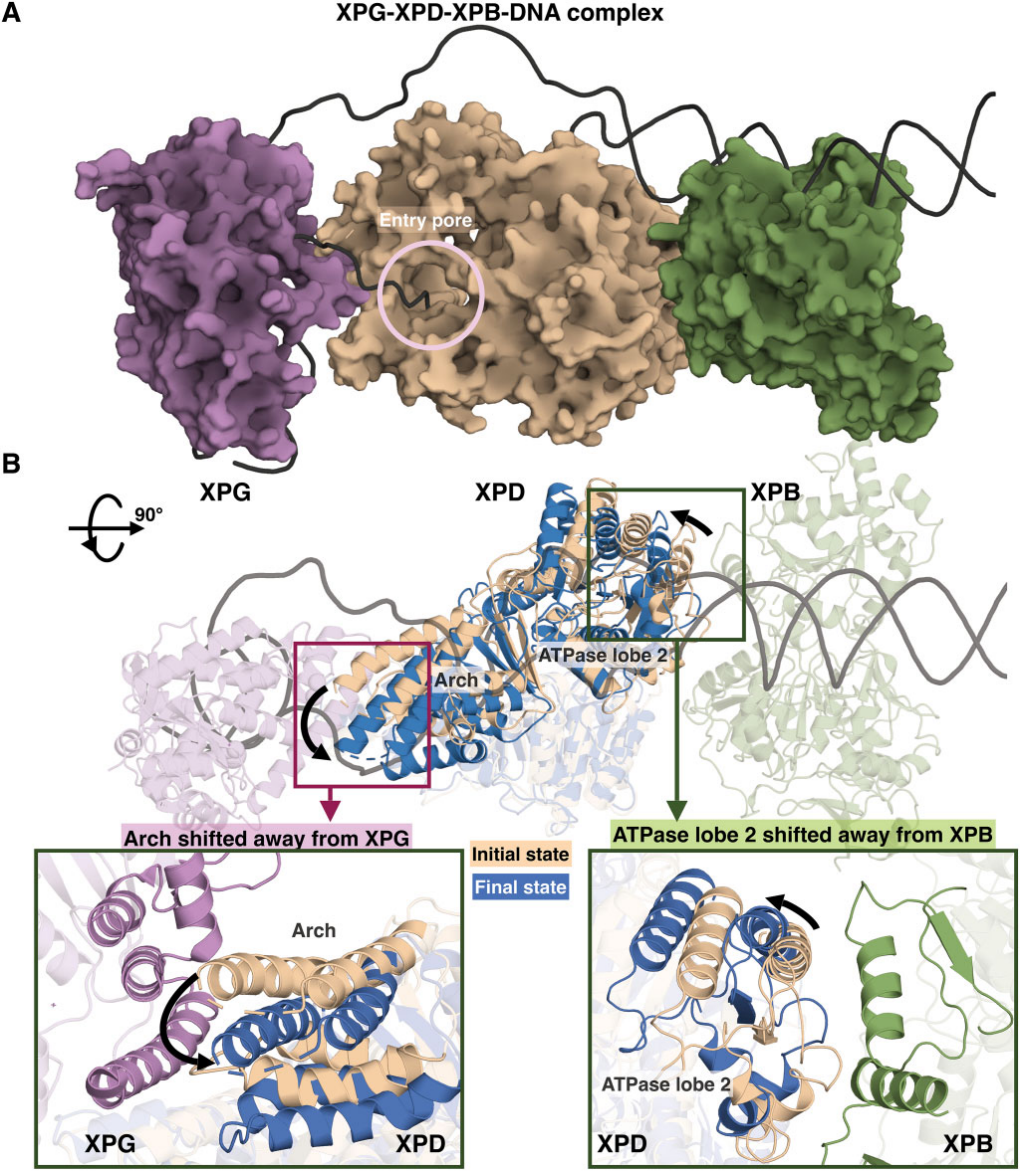
The displacement of the Arch domain changes the overall conformation of XPD within the complex of XPG-XPD-XPB bound to the open bubble substrate, altering direct XPG-XPD and XPD-XPB interactions, which could facilitate signaling for the subsequent incision step. (**A**) A model of XPG (purple), XPD (olive) and XPB (green) bound to the open bubble DNA (black), derived from the structural model provided by (45). In this model, we replaced the XPB and XPD helicases with those in the existing cryo-EM structure of TFIIH-XPA-DNA (44); XPG was replaced with the existing XPG crystal structure (PDB 3Q8K) (85). In this complex, XPD has direct contacts with XPG mainly via the Arch domain and direct contacts with XPB via the ATPase lobe 2 domain. (**B**) Superimposed structures of XPD prior to the shift of the Arch domain (olive) and after the shift of the Arch domain (blue) within the XPG-XPD-XPB-DNA complex highlight the changes in overall conformation of XPD, including the shift of the Arch domain away from XPG (left inset box) and the shift of the ATPase lobe 2 away from XPB (right inset box). Thus, it is likely that the excision process is helped by the change of overall XPD conformation in the pre-incision complex due to the lesion-imposed shift of the Arch domain that can be rapidly sensed by the XPG nuclease for the subsequent incision step.

We speculate that such a distinct displacement of the Arch domain that traps 6−4PP tightly within the tunnel may facilitate the repair of 6−4PP. In this tightly ‘trapped in the pore’ state, the perpendicular thymines of 6−4PP are clamped tightly between the Arch and the ATPase lobe1 domains and its backbone is anchored stably with the FeS domain. As a result, this trapped-in 6–4PP locks the Arch domain to the other two domains; this would inhibit the XPD helicase activity, as it would be unable to undergo the conformational change between the Arch and the FeS domains necessary to translocate DNA, as suggested by experimental studies (74,80). The Arch domain that undergoes thermally driven open-close transitions slightly favors the closed state with the presence of CPD (74), and locking the Arch domain to the FeS domain in a closed state inactivates the helicase activity of XPD (80). Furthermore, the XPD helicase activity is strongly stimulated by the presence of the XPG, highlighting the important role of the direct XPD-XPG interactions in regulating NER (44,45). Kokic *et al.* reported that DNA unwinding by XPD was 20-fold faster in the presence of XPG than in its absence (44). A recent single molecule imaging study by Bralic *et al.* (45) elucidated the mechanism by which XPG influences TFIIH activity and how TFIIH regulates 5′-incision by XPG; they uncovered a paired coordination between the core of TFIIH and XPG which characterizes lesion-scanning, efficient strand separation at the 3′ end of the NER bubble, and precise incision that occurs upon lesion detection. Thus, we reason that such a distinct displacement of the Arch domain toward only 6−4PP not only inhibits the XPD helicase activity but also facilitates the signaling to XPG for the subsequent incision step, fostered by the direct contact between XPD and XPG, which together facilitate the removal of 6−4PP. It is noteworthy that lesion recognition by XPD may not be sufficient for rapid excision of the damage-containing oligonucleotides. Perhaps this excision process is helped by the change in overall XPD conformation in the pre-incision complex due to the shift of the Arch domain that can be rapidly sensed by XPG nuclease for the subsequent incision step (Figure 5). Our findings are also consistent with experimental studies suggesting that differential repair of lesions may depend, at least in part, on differential helicase activity of XPD in NER: the dual-incision assay by Kim *et al.* (46) which showed that removal of the bulky lesion mimetic Cy5 fluorescent dye was more efficient than removal of AP sites in the presence of a seven-subunit TFIIH core (core7-TFIIH) bound to the damaged DNA, and the observations that mutations in the XPD gene in TTD cells affect much more dramatically the repair of CPD than 6−4PP (27,65,66).

In conclusion, we have obtained for the first time dynamic molecular characterizations of the differences in XPD’s inhibition by two topologically different UV lesions, CPD and 6−4PP via very extensive conformational sampling with long molecular dynamics simulations. We delineate lesion-dependent differences in atomistic and dynamic functioning of XPD in nucleotide excision repair, especially the roles of the Arch dynamics and lesion sensors in the dynamic mechanism of lesion verification. Furthermore, the presence of XPG has been shown to stimulate the XPD translocation activity (45), and XPG stabilizes the NER bubble which facilitates the first incision 5′ to the lesion by XPF-ERCC1 (48). These intriguing observations await further exploration to better delineate how XPD and XPG work together for lesion verification and repair.

## Supplementary Material

gkad974_Supplemental_Files

## Data Availability

The data underlying this article are available in the article and in its online supplementary material. The 8 μs MD simulation trajectories for XPD-64PP and XPD-CPD as well as the coordinates of the XPG-XPD-XPB-DNA model presented in Figure 5 are available in Figshare at https://doi.org/10.6084/m9.figshare.24201603.
