# Supplementary material for: Differing structures and dynamics of two photolesions portray verification differences by the human XPD helicase
Source: Nucleic Acids Res. 2023 Nov 2;51(22):12261–74. doi: 10.1093/nar/gkad974 (PMC10711554; doi:10.1093/nar/gkad974)
Supplement: gkad974_Supplemental_Files [file gkad974_supplemental_files.zip › NAR-SI-revision-final.pdf]

# Supplementary Data

## Differing structures and dynamics of two photolesions portray verification differences by the human XPD helicase

Iwen Fu[1], Nicholas E. Geacintov[2], and Suse Broyde[1]

[1]Department of Biology, New York University, 24 Waverly Place, 6th Floor, New York, NY, 10003, United States

[2]Department of Chemistry, New York University, 100 Washington Square East, New York, NY, 10003, United States

## Table of Contents

Supplementary Methods ........ 2

Force field ........ 2

Molecular dynamics simulations ........ 2

Supplementary Structural Analyses ........ 2

Key residues of the entry pore of XPD that encircles the nucleotides within ........ 3

Clustering analysis ........ 3

Stability of XPD and the lesion near the entry pore ........ 4

Analysis of hydrogen bonding between XPD and DNA ........ 4

Analysis of van der Waals interaction energy ........ 4

Supplementary Tables ........ 5

Supplementary Figures ........ 12

Supplementary Movies ........ 24

Supplementary References ........ 24

## Supplementary Methods

### *Force field*

We utilized the ff14SB [1] force field and the previously published parameters for the 6−4PP [2] and CPD[3] photolesions for the MD simulations. We employed the force field parameters published by Carvalho *et al*. [4] for the FeS cluster and TIP3P [5] water for the water molecules.

### *Molecular dynamics simulations*

All XPD-DNA complexes were explicitly solvated with TIP3P [5] water in a cubic periodic box with side length of 100 Å using the tLEAP module of the AMBER18 suite of programs [6]. Na+ ions were added to neutralize the system and 92 Na+/Cl− ion pairs were further added to reach the physiological salt concentration of ~ 150 mM. All systems were subject to energy minimization, equilibration and production runs using the same procedure as reported in our previous MD study [7]. These systems were first energy-minimized to remove close van der Waals contacts using SANDER of AMBER 18, which then was followed by equilibration and the classical NPT production runs of at least 8.0 µs using PMEMD of AMBER18 [6] with general-purpose graphics processing units (GPUs). All systems used the SHAKE-bond-length constraint applied to all bonds involving hydrogen and the particle-mesh Ewald (PME) [8, 9] method with 9.0 Å cutoff for the non-bonded interactions. The trajectories from the NPT production were saved every 10 ps for further structural and dynamic analysis. We excluded the highly mobile region of the Arch domain by applying an extra constraint between the backbone V270 C and A326 C atoms during the equilibration and production MD runs to stabilize these two ends, which connect the plug of the Arch domain.

## Supplementary Structural Analyses

Post-processing and analysis of all simulations was performed using the CPPTRAJ [10] module of AMBER18 [6]. All mean values and standard deviations for the stable ensemble of each MD simulation were computed using the block averaging method [11, 12]. Molecular images and movies were generated with PyMOL (Schrodinger, LLC. [13]) and VMD [14].

### *Key residues of the entry pore of XPD that encircles the nucleotides within*

In the cryo-EM structure of XPD–ssDNA complex with PBD ID 6RO4 [15], the last two nucleotides (3'dT and dA0) at the 3'-end of the DNA are encircled by the XPD helices (see figure right), including the FeS residues 109 – 112, 128–138, 190 – 200, the Arch residues 373–409, and the ATPase lobe 1 residues 215 – 221. Thus, we utilized these helices to delineate the DNA entry pore of XPD.

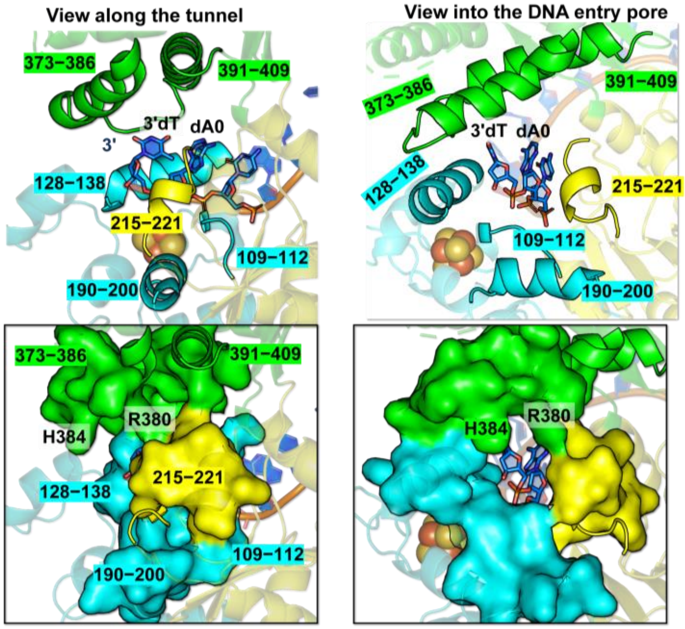


### *Clustering analysis*

We utilized the last 2 μs for the ensemble analyses to capture the properties of the equilibrated states for all MD simulations. The most representative structures of the XPD-DNA complexes from the equilibrated states were obtained based on the local region near the entry pore (see figure below) using cluster analysis, which was performed using the average linkage hierarchical agglomerative method [16] and RMSD as the distance matrix. XPD of this local region includes the ATPase lobe 1 residues 69–88, 104–109, 204–226, 239–247, the FeS residues 110–138, 154–164, 177–200, and the Arch residues 369–409 (as indicated in the black oval in the left panel of the figure); the DNA of this local region includes the lesion and its 5'-dA (see right panel).

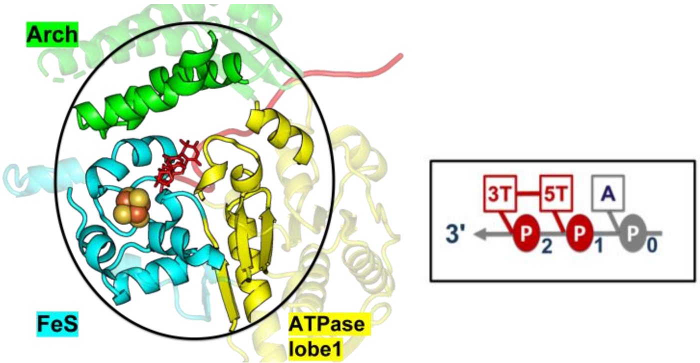


**Left panel: the local region near the DNA entry pore considered for clustering.**
**Right panel: the DNA near the pore used for clustering.**

***Stability of XPD and the lesion near the entry pore***

We computed the time-dependent backbone Cα RMSDs of the XPD pore as indicated in the black oval (figure above) to estimate its stability in each XPD-CPD (**Supplementary Figure S1**) and XPD-64PP simulation (**Supplementary Figure S2**). We also computed the time-dependent heavy atom and backbone P atom RMSDs of each lesion in the lesion-containing XPDs.

***Analysis of hydrogen bonding between XPD and DNA***

Hydrogen bonds of the DNA near the pore with surrounding XPD residues were computed for the equilibrium ensemble in each XPD-ssDNA simulation. Hydrogen bonds were counted using the criterion for each hydrogen bond pair (donor-acceptor pair) when the hydrogen bond distance (donor-to-acceptor atom) is ≤ 3.5Å and the hydrogen bond angle (donor-hydrogen-acceptor) is ≥ 120°. A fractional hydrogen bond between donor and acceptor is one that is present in the corresponding fraction of the population of the analyzed MD trajectories.

***Analysis of van der Waals interaction energy***

We computed the van der Waals interaction energies of the individual domains near the entry pore, including the ATPase lobe 1 residues 215−221, the Arch residues 369−409, and the FeS residues 128−200, with the modified bases of the lesion in XPD-CPD (**Supplementary Tables S1-S2**) and in XPD-64PP (**Supplementary Tables S4-S5**), using the CPPTRAJ [10] module of AMBER18 [6] for the Lennard-Jones potential.

## Supplementary Tables

**Supplementary Table S1**. The XPD-lesion interactions corresponding to each of the ten independent simulations (S1 − S10) of XPD-CPD-L. (A) Best representative structures are shown (see Supplementary Structural Analyses). (B) Van der Waals interaction energies between the modified bases of the lesion and the XPD (Arch, FeS, ATPase lobe1). Mean values and standard deviations are listed. (C) Number of hydrogen bonds between lesion (backbone: P, PB; Bases) and individual residue/XPD are listed in black/red. The 5'-phosphate and the crosslinked-phosphate atoms of the lesion are labelled as P and PB, respectively. Individual hydrogen bond pairs between the bases of CPD and the surrounding XPD residues are listed in **Supplementary Table S3.**

**A** XPD-CPD-L

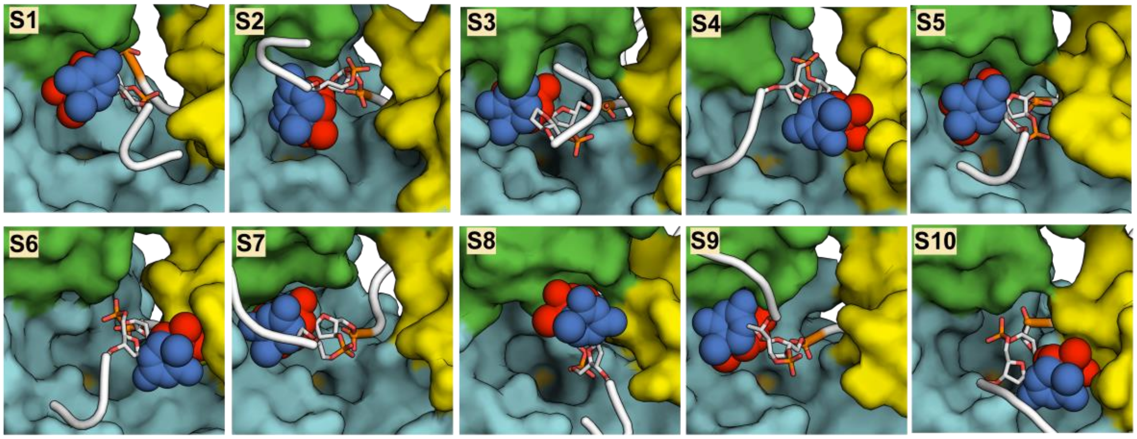


**B** vdW interactions (kcal/mol) between lesion (Bases) and XPD (Arch, FeS, ATPase lobe 1)

| | S1 | S2 | S3 | S4 | S5 | S6 | S7 | S8 | S9 | S10 |
|---|---|---|---|---|---|---|---|---|---|---|
| **XPD** | **−9.80 (± 0.45)** | **−9.77 (±0.25)** | **−14.16 (± 0.48)** | **−9.10 (± 0.36)** | **−10.58 (± 0.31)** | **−10.41 (± 1.01)** | **−16.30 (± 0.80)** | **−12.73 (± 0.39)** | **−16.50 (± 0.43)** | **−7.55 (± 1.03)** |
| **FeS** | −3.97 (± 0.20) | −8.37 (±0.23) | −11.68 (± 0.39) | −0.29 (± 0.13) | −6.67 (± 0.15) | −0.48 (± 0.18) | −11.36 (± 0.41) | 0 | −11.70 (± 0.33) | −1.65 (± 0.55) |
| **Arch** | −5.83 (± 0.64) | −1.35 (±0.09) | −2.48 (± 0.37) | 0 | −3.88 (± 0.26) | 0 | −4.94 (± 0.68) | −10.98 (± 0.29) | −4.81 (± 0.19) | 0 |
| **ATPase Lobe 1** | 0 | 0 | 0 | −8.72 (± 0.27) | 0 | −9.93 (± 1.02) | 0 | −1.71 (± 0.24) | 0 | −5.88 (± 0.77) |

**C** Number of HBs between lesion (P, PB, Bases) and surrounding residues

| | S1 | S2 | S3 | S4 | S5 | S6 | S7 | S8 | S9 | S10 |
|---|---|---|---|---|---|---|---|---|---|---|
| **P** | **1.94** | **3.17** | **2.88** | **4.75** | **4.26** | **4.8** | **3.25** | **4.27** | **3.80** | **3.60** |
| **R112** | | 1.71 | | 1.89 | 1.99 | 1.98 | 1.76 | 2.40 | 2.00 | 1,32 |
| **R196** | | 1.46 | 2.22 | 2.86 | 2.17 | 2.83 | 1.49 | 1.87 | 1.80 | 2.28 |
| **K128** | 0.52 | | | | | | | | | |
| **R380** | 1.47 | | 0.66 | | 0.11 | | | | | |
| **PB** | **0** | **2.80** | **0.47** | **0.72** | **1.48** | **1,89** | **0** | **0.29** | **0** | **1.45** |
| **R380** | | 2.80 | | 0.72 | | 1.89 | | | | 1.45 |
| **R196** | | | 0.47 | | 0.78 | | | 0.29 | | |
| **K223** | | | | | 0.70 | | | | | |
| **Bases** | **1.53** | **0.93** | **1.33** | **1.88** | **2.37** | **2.70** | **2.84** | **0.13** | **1.66** | **2.11** |

**Supplementary Table S2**. The XPD-lesion interactions corresponding to each of the ten independent simulations (S11 – S20) of XPD-CPD-R. (**A**) Best representative structures. (**B**) Van der Waals interaction energies between the modified bases of the lesion and the XPD (Arch, FeS, ATPase lobe1). Mean values and standard deviations are listed. (C) Number of hydrogen bonds between lesion (backbone: P, PB; Bases) and individual residue/XPD are listed as **black/red**. The 5'-phosphate and the crosslinked-phosphate atoms of the lesion are labelled as P and PB, respectively. Individual hydrogen bond pairs between the bases of CPD and the surrounding XPD residues are listed in **Supplementary Table S3**.

**A** XPD-CPD-R

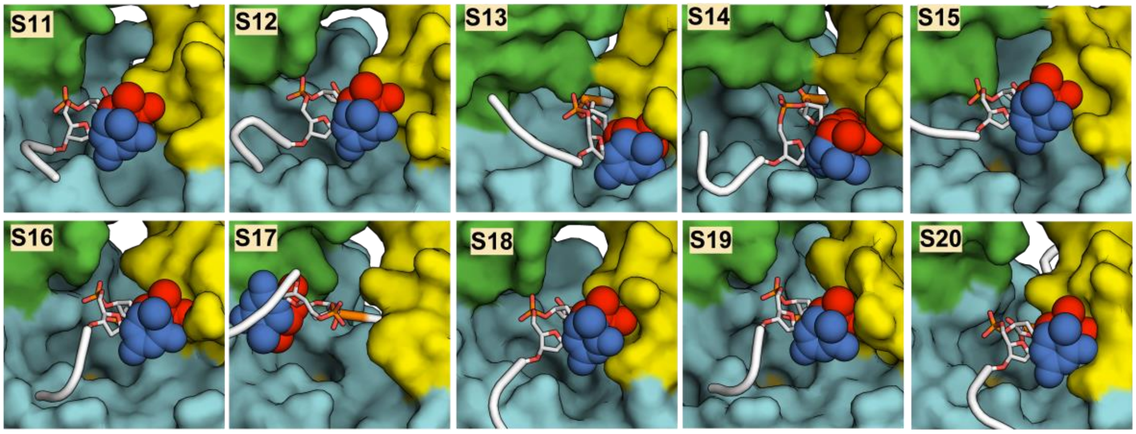


**B** vdW interactions (kcal/mol) between lesion (Bases) and XPD (Arch, FeS, ATPase lobe 1)

| | S11 | S12 | S13 | S14 | S15 | S16 | S17 | S18 | S19 | S20 |
|---|---|---|---|---|---|---|---|---|---|---|
| XPD | −8.04 (± 0.69) | −6.03 (±0.22) | −16.24 (± 2.61) | −14.02 (± 0.78) | −6.36 (± 0.29) | −10.00 (± 0.36) | −14.09 (± 0.87) | −8.15 (± 0.71) | −10.09 (± 0.61) | −13.34 (± 0.82) |
| FeS | −2.55 (± 0.16) | −0.63 (±0.34) | −8.44 (± 2.33) | −7.61 (± 0.56) | −0.83 (± 0.17) | −0.50 (± 0.20) | −8.30 (± 0.77) | −0.88 (± 0.20) | −0.57 (± 0.19) | −1.35 (± 0.39) |
| Arch | 0 | 0 | 0 | 0 | 0 | 0 | −5.77 (± 0.69) | 0 | 0 | 0 |
| ATPase Lobe 1 | −5.46 (± 076) | −5.37 (±0.3)) | −7.78 (± 0.54) | −6.36 (± 0.37) | −5.46 (± 0.28) | −9.50 (± 0.44) | 0 | −7.23 (± 0.90) | −9.52 (± 0.72) | −11.99 (± 0.95) |

**C** Number of HBs between lesion (P, PB, Bases) and surrounding residues

| | S11 | S12 | S13 | S14 | S15 | S16 | S17 | S18 | S19 | S20 |
|---|---|---|---|---|---|---|---|---|---|---|
| **P** | **4.26** | **4.16** | **3.02** | **3.77** | **4.48** | **4.76** | **3.88** | **4.27** | **4.82** | **4.55** |
| R112 | 1.97 | 1.92 | | | 1.91 | 1.93 | 1.39 | 1.88 | 1.97 | 1.95 |
| R196 | 2.29 | 2.24 | 2.50 | 2.32 | 2.43 | 2.83 | 1.73 | 2.39 | 2.85 | 2.60 |
| K128 | | | | | | | 0.76 | | | |
| R380 | | | 0.52 | 1.45 | 0.14 | | | | | |
| **PB** | **1.52** | **1.53** | **0.92** | **1.73** | **1,91** | **2.09** | **0** | **1.50** | **2.35** | **0** |
| R380 | 1.52 | 1.53 | 0.92 | 1.73 | 1.91 | 2.09 | | 1.50 | 2.35 | |
| **Bases** | **2.43** | **1.89** | **2.27** | **3.00** | **1.99** | **2.52** | **3.02** | **2.60** | **2.46** | **3.93** |

**Supplementary Table S3.** Individual hydrogen bond pairs between the modified bases and the surrounding XPD residues near the pore in the 20 simulations (S1, S2, S3…,S20) for CPD. Individual hydrogen bond pairs (atom names A-B are given; A is for XPD and B for lesion) between the residues and modified bases as well as the corresponding fractional hydrogen bonds (if greater than 0.1) are listed. A fractional hydrogen bond is one that is present in the corresponding fraction of the population of the analyzed MD trajectories. Total hydrogen bond numbers (Total HBs) of each XPD residue-CPD are also listed (shown in red).

**XPD-CPD**

| | S1 | S2 | S3 | S4 | S5 | S6 | S7 | S8 | S9 | S10 |
|---|---|---|---|---|---|---|---|---|---|---|
| R380 | NH1-O2<br>0.43<br>NE-O2T<br>0.12 | | | | NE-O2<br>0.96<br>NH2-O2<br>0.41 | | NH1-O2T<br>0.67<br>NH1-O2<br>0.46 | | NH1-O2T<br>0.12<br>NH2-O2<br>0.15 | |
| H384 | NE2-N3<br>0.98 | | ND1-O2T<br>0.59<br>ND1-N3<br>0.10 | | NE2-N3<br>0.99 | | NE2-N3<br>0.50<br>NE2-N3T<br>0.34 | | NE2-N3T<br>0.73<br>ND1-N3T<br>0.10 | |
| E377 | | | | | | | | OE2-N3T<br>0.13 | | |
| H135 | | NE2-N3T<br>0.97 | | | | | | | ND1-N3<br>0.19 | |
| S140 | | | N-O4T<br>0.65 | | | | N-O4T<br>0.84 | | N-O4T<br>0.38 | |
| R166 | | | | | | | | | | NH1-O2T<br>0.44 |
| R196 | | | | NH2-O2<br>0.94 | | NH2-O2<br>0.98 | | | | NH2-O2<br>0.88<br>NE-O2<br>0.21 |
| L220 | | | | O-N3<br>0.12 | | | | | | O-N3<br>0.59 |
| V221 | | | | | | O-N3<br>0.81 | | | | |
| S222 | | | | OG-O4<br>0.72 | | | | | | |
| K223 | | | | NZ-O4T<br>0.10 | | N-O4<br>0.15 | | | | |
| E224 | | | | | | N-O4<br>0.75 | | | | |
| Total HBs | 1.53 | 0.93 | 1.33 | 1.88 | 2.37 | 2.70 | 2.84 | 0.13 | 1.66 | 2.11 |

| | S11 | S12 | S13 | S14 | S15 | S16 | S17 | S18 | S19 | S20 |
|---|---|---|---|---|---|---|---|---|---|---|
| R380 | | | | | | | NH1-O2<br>0.13<br>NH2-O2<br>0.22<br>NE-O2<br>0.28<br>NH1-O2T<br>0.23 | | | |
| H384 | | | | | | | NE2-N3<br>0.83<br>NE2-N3T<br>0.15 | | | |
| S140 | | | | | | | N-O4T<br>0.84<br>OG-O4T<br>0.33 | | | |
| R166 | NH1-O2T<br>0.64 | | | | | | | | NH1-O2T<br>0.49 | |
| R196 | NH1-O2<br>1.00 | NH2-O2<br>0.97 | NH2-O2<br>0.61<br>NE-O2<br>0.14<br>O-N3T<br>0.40<br>O-N3<br>0.31 | NH2-O2<br>1.00<br>NE-O2<br>0.40<br>O-N3T<br>0.98<br>O-N3T<br>0.98 | NH2-O2<br>0.96 | NH2-O2<br>0.98 | | NH2-O2<br>0.95 | NH2-O2<br>0.96 | NH2-O2<br>0.98<br>NE-O2<br>0.16<br>NH1-O2T<br>0.12 |
| Y197 | | | O-N3T<br>0.16 | | | | | | | |
| L220 | O--N3<br>0.69 | O-N3<br>0.92 | | | O-N3<br>0.92 | | | O-N3<br>0.87 | | |
| V221 | | | O-N3<br>0.43 | O-N3<br>0.59 | | O-N3<br>0.80 | | | O-N3<br>0.71 | O-N3<br>0.65 |
| K223 | NZ-O4T<br>0.10 | | N-O4<br>0.21 | | NZ-O4<br>0.10 | | | | N-O4<br>0.11 | N-O4<br>0.49 |
| E224 | | | | | | N-O4<br>0.73 | | OE1-N3T<br>0.41<br>OE2-N3T<br>0.38 | N-O4<br>0.68 | N-O4<br>0.65<br>OE1-N3T<br>0.40<br>OE2-N3<br>0.49 |
| Total HBs | 2.43 | 1.89 | 2.27 | 3.00 | 1.99 | 2.52 | 3.02 | 2.60 | 2.46 | 3.93 |

**Supplementary Table S4**. The XPD-lesion interactions corresponding to each of the ten independent simulations (S1 – S10) of XPD-64PP-L. (**A**) Best representative structures. (**B**) Van der Waals interaction energies between the modified bases of the lesion and the XPD (Arch, FeS, ATPase lobe1). Mean values and standard deviations are listed. (C) Hydrogen bond numbers between lesion (backbone: P, PB; Bases) and individual residue/XPD are listed as **black**/**red**. The 5'-phosphate and the crosslinked-phosphate atoms of the lesion are labelled as P and PB, respectively. Note that individual hydrogen bond pairs between the bases of CPD and the surrounding XPD residues are listed in **Supplementary Table S6**.

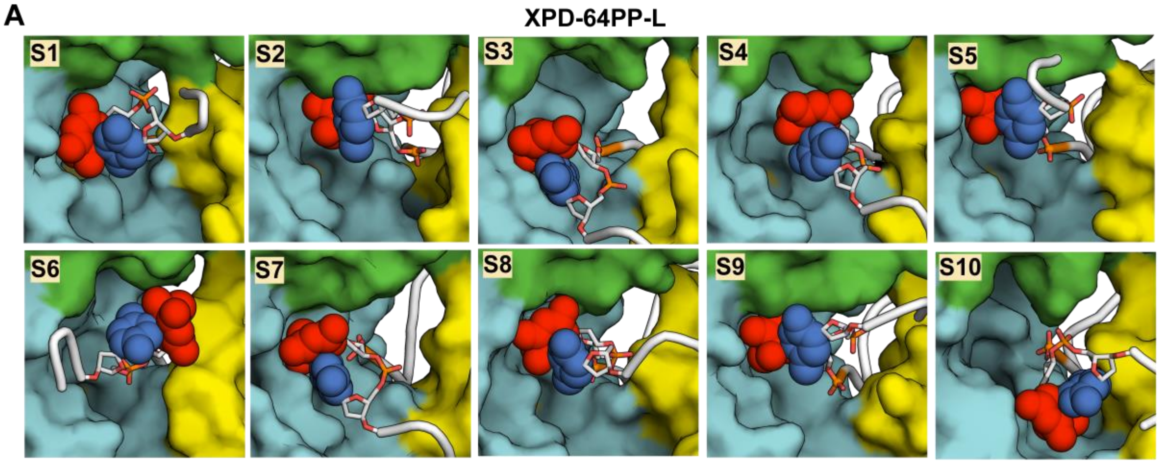


**B**

vdW interactions (kcal/mol) between lesion (Bases) and XPD (Arch, FeS, ATPase lobe 1)

| | S1 | S2 | S3 | S4 | S5 | S6 | S7 | S8 | S9 | S10 |
|---|---|---|---|---|---|---|---|---|---|---|
| **XPD** | **−9.60 (± 0.21)** | **−10.96 (±0.89)** | **−10.62 (± 0.63)** | **−4.62 (± 0.36)** | **−13.17 (± 0.53)** | **−5.74 (± 0.45)** | **−12.21 (± 0.43)** | **−8.52 (± 0.50)** | **−12.21 (± 0.43)** | **−9.74 (± 0.23)** |
| **FeS** | −8.26 (± 0.11) | −6.39 (±0.18) | −9.77 (± 0.78) | −`1.20 (± 0.22) | −8.33 (± 0.29) | −0.45 (± 0.19) | −10.32 (± 0.56) | −5.47 (± 0.88) | −7.57 (± 0.46) | −7.46 (± 0.25) |
| **Arch** | −1.10 (± 0.12) | −4.56 (±0.96) | −0.84 (± 0.46) | −3.34 (± 0.33) | −4.79 (± 0.39) | −2.61 (± 0.36) | −1.89 (± 0.35) | −3.02 (± 0.84) | −4.62 (± 0.62) | 0) |
| **ATPase Lobe 1** | 0 | 0 | 0 | 0 | 0 | −2.67 (± 0.50) | 0 | 0 | 0 | −2.26 (± 0.09) |

**C**

Number of HBs between lesion (P, PB, Bases) and surrounding residues

| | S1 | S2 | S3 | S4 | S5 | S6 | S7 | S8 | S9 | S10 |
|---|---|---|---|---|---|---|---|---|---|---|
| **P** | **4.36** | **2.54** | **2.62** | **3.58** | **3.55** | **3.70** | **2.51** | **3.27** | **2.15** | **3.76** |
| **R112** | 1.87 | 0 | 2.62 | 2.43 | 2.00 | 1.49 | 1.95 | 2.00 | | 1.82 |
| **R196** | 2.49 | 2.54 | | 1.15 | 1.55 | 2.21 | | 1.26 | 2.15 | 1.94 |
| **R380** | | | | | | | 0.56 | | | |
| **PB** | **0.99** | **0** | **2.22** | **0.85** | **0.87** | **0.35** | **0** | **0.40** | **0.29** | **2.0** |
| **R196** | | | 2.00 | 0.85 | | 0.15 | | | | |
| **K216** | 0.99 | | | | 0.66 | 0.20 | | 0.10 | | |
| **K223** | | | 0.22 | | | | | | | |
| **R380** | | | | | 0.20 | | | 0.30 | 0.29 | 2.0 |
| **Bases** | **0.30** | **1.00** | **1.82** | **0** | **1.56** | **0.65** | **1.61** | **1.10** | **1.12** | **0.23** |

**Supplementary Table S5**. The XPD-lesion interactions corresponding to each of the ten independent simulations (S11 – S20) of XPD-64PP-R. (**A**) Best representative structures. (**B**) Van der Waals interaction energies between the modified bases of the lesion and the XPD (Arch, FeS, ATPase lobe1). Mean values and standard deviations are listed. (C) Hydrogen bond numbers between lesion (backbone: P, PB; Bases) and individual residue/XPD are listed as black/red. The 5'-phosphate and the crosslinked-phosphate atoms of the lesion are labelled as P and PB, respectively. Individual hydrogen bond pairs between the bases of CPD and the surrounding XPD residues are listed in **Supplementary Table S6**.

**A** XPD-64PP-R

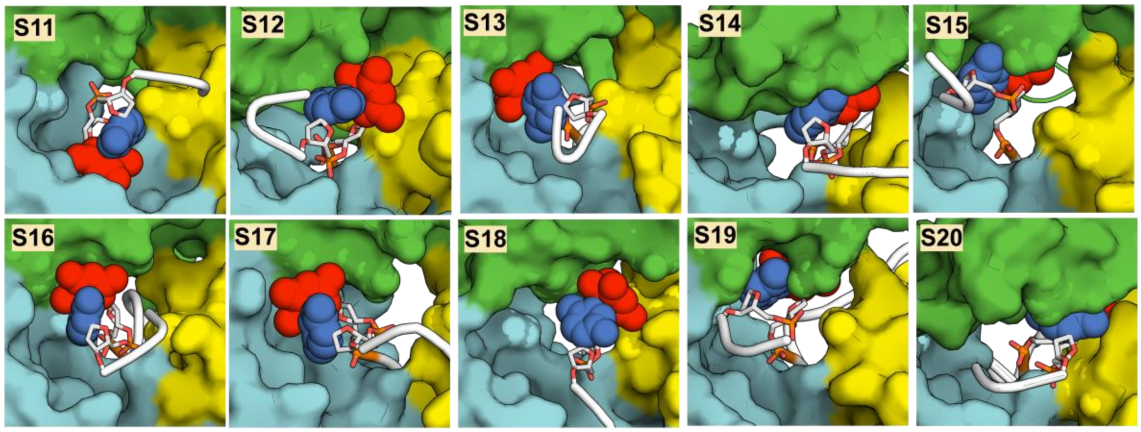


**B** vdW interactions (kcal/mol) between lesion (Bases) and XPD (Arch, FeS, ATPase lobe 1)

| | S11 | S12 | S13 | S14 | S15 | S16 | S17 | S18 | S19 | S20 |
|---|---|---|---|---|---|---|---|---|---|---|
| **XPD** | **−8.62 (± 0.53)** | **−5.67 (±0.50)** | **−9.75 (± 0.35)** | **−10.28 (± 0.48)** | **−11.31 (± 0.40)** | **−8.68 (± 0.89)** | **−10.12 (± 0.39)** | **−8.34 (± 0.57)** | **−9.80 (± 0.42)** | **−16.66 (± 0.31)** |
| **FeS** | −7.92 (± 0.48) | 0 | −4.67 (± 0.32) | −0.22 (± 0.05) | −2.55 (± 0.40) | −1.77 (± 0.39) | −5.95 (± 0.91) | 0 | −1.33 (± 0.21) | 0 |
| **Arch** | 0 | −1.52 (±0.55) | −5.06 (± 0.34) | −8.19 (± 0.51) | −8.10 (± 0.33) | −6.87 (± 0.76) | −4.17 (± 0.51) | −5.93 (± 0.51) | −7.98 (± 0.59) | −8.01 (± 0.39) |
| **ATPase Lobe 1** | −0.62 (±0.12) | −4.08 (±0.54) | 0 | −1.87 (± 0.22) | −0.66 (± 0.11) | 0 | 0 | −2.31 (± 0.42) | −0.48 (± 0.05) | −8.62 (± 0.19) |

**C** Number of HBs between lesion (P, PB, Bases) and surrounding residues

| | S11 | S12 | S13 | S14 | S15 | S16 | S17 | S18 | S19 | S20 |
|---|---|---|---|---|---|---|---|---|---|---|
| **P** | **4.6** | **5.74** | **3.10** | **1.25** | **4.17** | **2.54** | **2.61** | **3.26** | **4.12** | **2.00** |
| **R112** | 2.00 | 1.81 | 2.20 | | 1.96 | 2.54 | 2.17 | 1.15 | 1.94 | |
| **R196** | 2.60 | 2.45 | 0.90 | 1.25 | 2.21 | | 0.44 | 1.97 | 2.18 | 2.00 |
| **R380** | | 1.48 | | | | | | 0.14 | | |
| **PB** | **1.44** | **0** | **0.34** | **2.13** | **0.87** | **2.96** | **0.95** | **1.42** | **0.90** | **0.85** |
| **R196** | | | | 2.13 | 0.87 | 2.61 | | 1.42 | 0.90 | 0.85 |
| **K223** | | 0.11 | | | | | | | | |
| **R380** | 1.44 | | | | | 0.35 | 0.95 | | | |
| **E377** | | 0.96 | | | | | | | | |
| **Bases** | **0.93** | **1.07** | **0.36** | **0.85** | **1.40** | **1.29** | **0.31** | **0.64** | **1.12** | **2.43** |

**Supplementary Table S6**. Individual hydrogen bond pairs between the modified bases and the surrounding XPD residues near the pore in the 20 simulations (S1, S2, S3…,S20) for 6−4PP. Individual hydrogen bond pairs (atom names A-B are given; A is for XPD and B for lesion) between the residues and modified bases as well as the corresponding fractional hydrogen bonds (if greater than 0.1) are listed. A fractional hydrogen bond is one that is present in the corresponding fraction of the population of the analyzed MD trajectories. Total hydrogen bond numbers (Total HBs) of each XPD residue-lesion are also listed (red).

**A** XPD-64PP

| | S1 | S2 | S3 | S4 | S5 | S6 | S7 | S8 | S9 | S10 |
|---|---|---|---|---|---|---|---|---|---|---|
| R380 | | NE-O2T 0.76 NH2-O2T 0.12 | | | NE-O2T 0.50 | NE-O4T 0.11 | NH1-O2 0.13 | NH1-O2 0.36 | NE-O2T 0.32 | |
| H384 | | | | | NE2-N3 0.87 | | | NE2-NE2 0.25 | NE2-N3 0.80 | |
| E377 | | | | | | OE1-O4T 0.19 OE2-O4T 0.18 | | | | |
| H135 | | ND1-N3 0.12 | | | | | | | | |
| T138 | OH-O4T 0.20 | | O-O4T 0.77 | | | | O-O4T 0.57 | | | |
| S140 | N-O4 0.10 | | N-O4 0.36 | | N-O4 0.19 | | N-O4 0.44 | N-O4 0.49 | | |
| Y158 | | | OH-O4T 0.69 | | | | OH-O4T 0.47 | | | |
| R166 | | | | | | | | | | NH2-O2 0.23 |
| K223 | | | | | | NZ-O2 0.17 | | | | |
| Total HBs | 0.30 | 1.00 | 1.82 | 0 | 1.56 | 0.65 | 1.61 | 1.10 | 1.12 | 0.23 |

**B**

| | S11 | S12 | S13 | S14 | S15 | S16 | S17 | S18 | S19 | S20 |
|---|---|---|---|---|---|---|---|---|---|---|
| R380 | | | NE-O2 0.21 NH1-O2 0.15 | NH1-O2T 0.85 | NH1-O2T 1.00 NH1-N3T 0.16 | NE-O2 0.24 | | | NH1-O2T 1.00 NH1-N3T 0.12 | NH1-N3T 0.71 NH1-O2T 0.64 |
| S381 | | | | | | | | OG-O4T 0.27 | | |
| H384 | | | | | | NE2-O4T 0.57 | | | | |
| E377 | | OE2-O4T 0.49 OE1-O4T 0.47 | | | | OE1-N3 0.25 OE2-N3 0.23 | | | | |
| N402 | | | | | ND2-O4 0.24 | | | | | |
| S140 | | | | | | | N-O4 0.31 | | | |
| Y158 | OH-O2 0.93 | | | | | | | | | |
| D219 | | | | | | | | | | N-O2 0.77 OD1-N3 0.17 OD2-N3 0.14 |
| K223 | | NZ-O2 0.11 | | | | | | NZ-O2 0.38 | | |
| Total HBs | 0.93 | 1.07 | 0.36 | 0.85 | 1.40 | 1.29 | 0.31 | 0.64 | 1.12 | 2.43 |

## Supplementary Figures

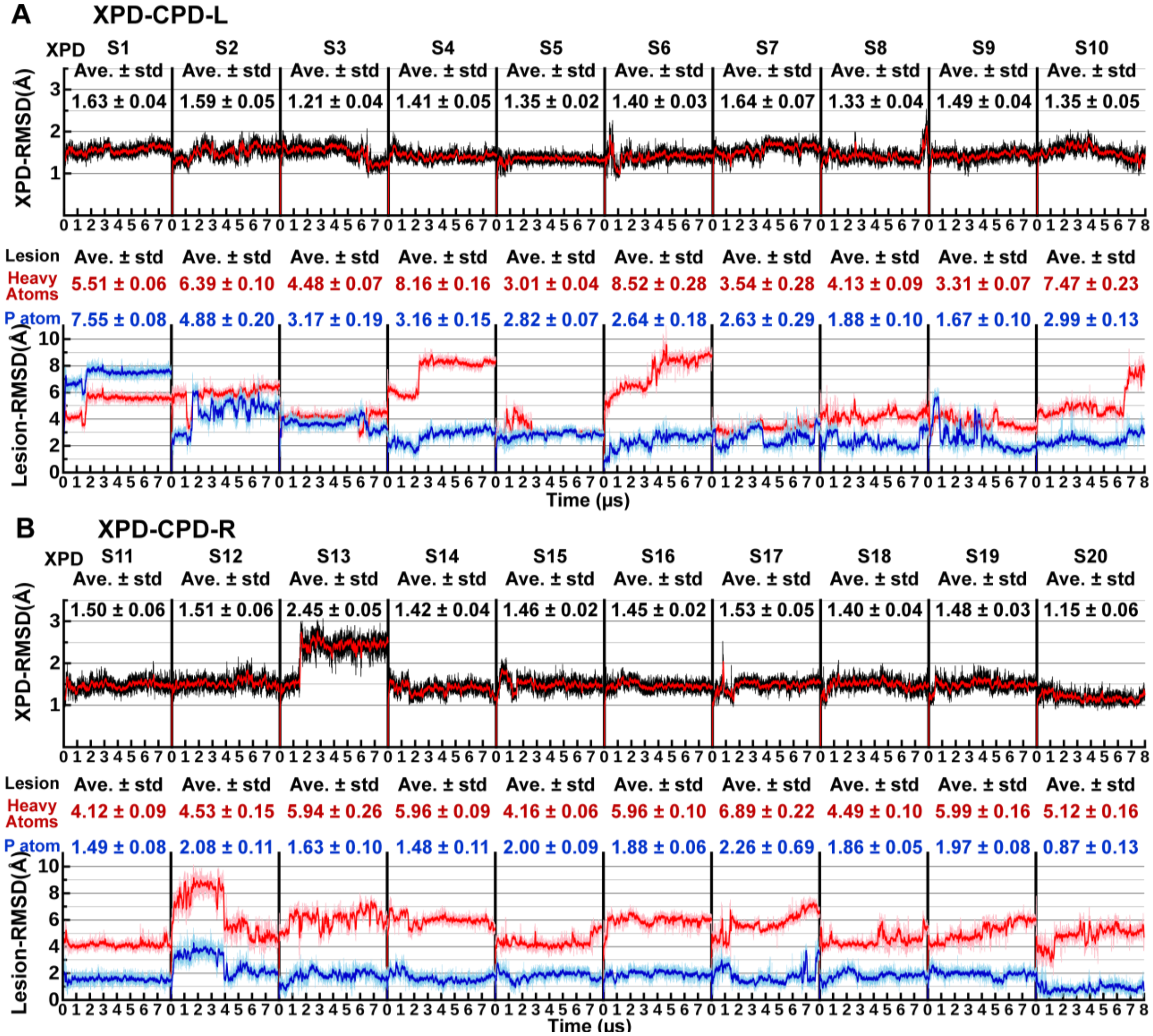


**Supplementary Figure S1.** XPD retains a correctly folded pore when binding to CPD-containing ssDNA in the 20 independent MD simulations of ~ 8 μs each for each initial structure (A) S1−S10 of XPD-CPD-L and (B) S11 – S20 of XPD-CPD-R.

Here we computed Cα RMSDs (black) of the XPD pore as well as the RMSDs of heavy-atoms (red) and the backbone P atom (blue) of the lesion while superimposing the XPD pore for all 20 simulations. In these simulations, the time-dependent Cα RMSDs of the XPD pore (black line) reveal that the XPD pore reaches a stable state after ~ 1.7 μs and retains a correctly folded structure with RMSDs of ~ 1.1 − 1.7 Å from its initial fold (except S13, see **Supplementary Figure S3**). As the extended DNA containing a CPD lesion outside the entry pore is generally flexible, the lesion cases take longer and with varying amounts of time to reach an equilibrated state. We utilized the last 2 μs of each simulation for the equilibrium ensemble analyses. Mean values and standard deviations of the corresponding RMSDs for the XPD pore and the lesion for each simulation are given.

The CPD lesion positioned outside the entry pore displays varying deviations from its initial state, with ensemble average heavy-atom RMSD values ranging from 3 to 9 Å. Notably, in one of the 20 simulations (see S1), the backbone of CPD near the entry pore exhibits a large displacement from its initial position; this displacement is dominated by backbone 3'→5' movement manifested by P atom RMSD of 7.55 ± 0.08 Å.

In other simulations, the phosphate backbone of the CPD lesion is well-anchored to the FeS residues R112 or R196 or both (**Supplementary Tables S1C and S2C**); its phosphate backbone remains rather close to its initial position near the entry pore, reflected in the low values of the P atom RMSDs of ~ 0.8 − 4.8 Å. Thus, the deviation of the lesion is mainly due to the reorientation of the modified bases from their initial positions, with varying orientations of CPD outside the entry pore (see **Supplementary Tables S1A and S2A**).

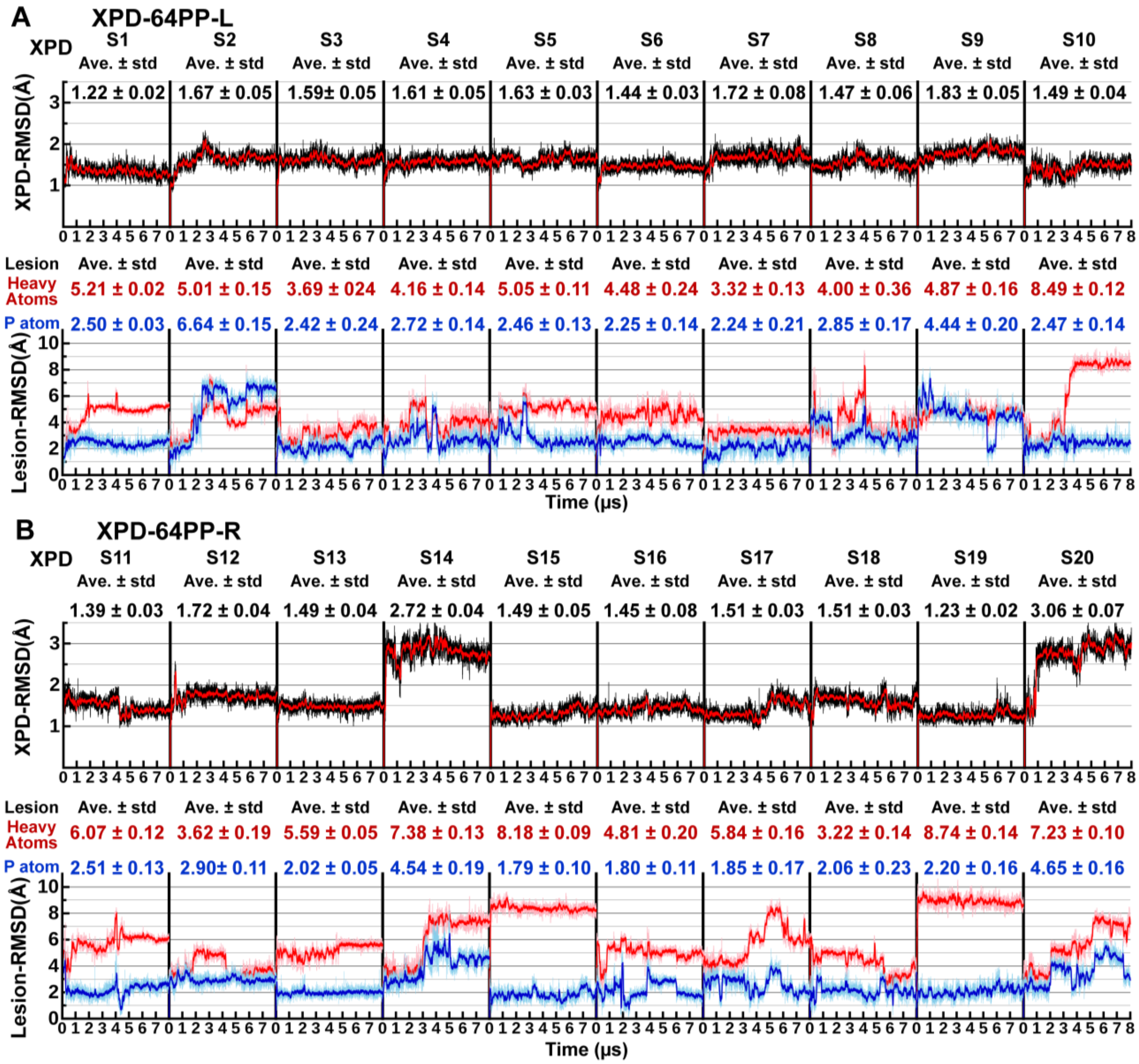


**Supplementary Figure S2.** The XPD mainly retains a crystal-like folded pore when binding to ssDNA containing the 6−4PP lesion outside the entry pore but deviates in some cases. (A) S1−S10 of XPD-64PP-L and (B) S11 – S20 of XPD-64PP-R.

In most simulations, the XPD retains a correctly and stably folded structure with RMSDs of ~ 1.2 – 1.8 Å from its initial fold. However, in 2 of 20 simulations, S14 and S20, of XPD-64PP, the XPD fold shows significant deviations with RMSDs values up to 3Å from its initial state, and the largest deviation is seen in S20 of the XPD-64PP case. This deviation of the XPD fold mainly stems from the displacement of the Arch domain with respect to the FeS domain (**Supplementary Figure S3**).

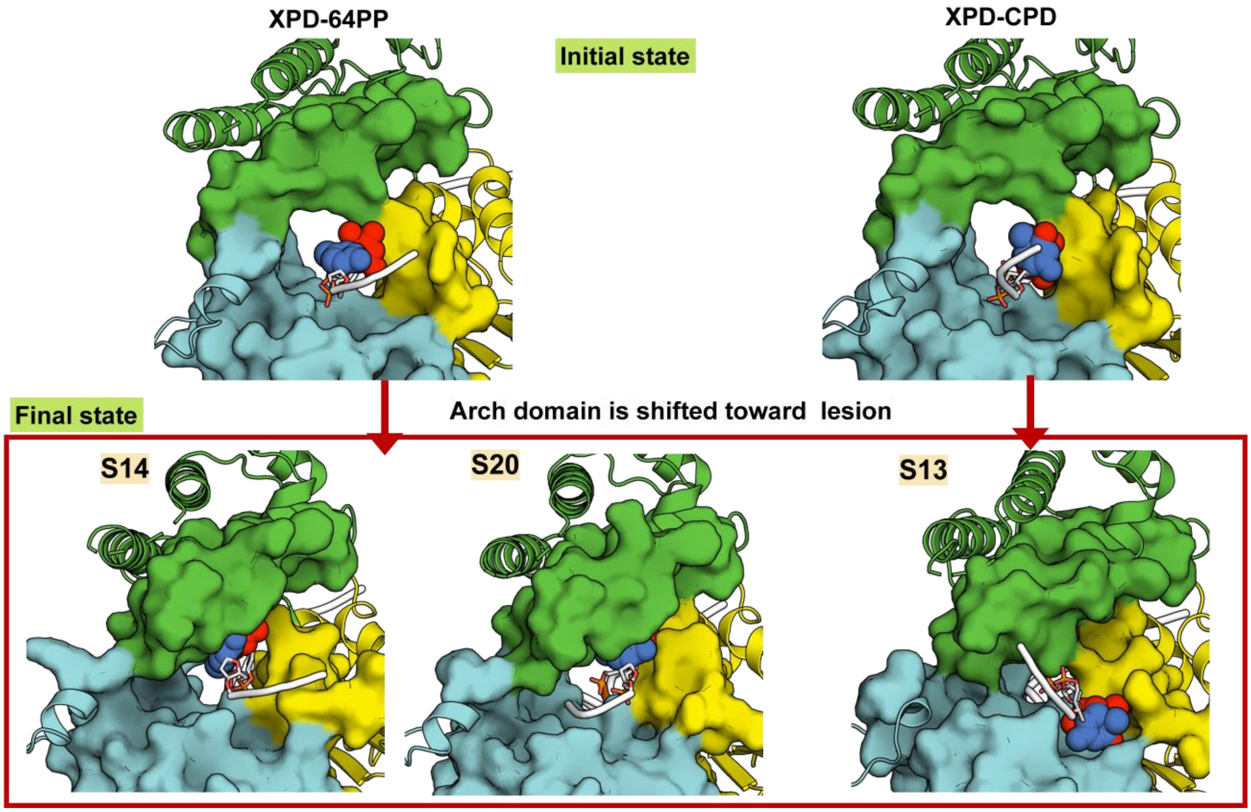


**Supplementary Figure S3.** The Arch domain can be shifted toward the lesion and the ATPase lobe 1 domain when encountering a lesion near the pore.

The Arch domain can be shifted from its initial fold toward the lesion and the ATPase lobe 1 domain when the lesion is pointed toward ATPase lobe 1, shrinking the size of the pore. Thus, the Arch and ATPase lobe 1 domains can then hold the lesion more tightly, as shown in the cases of XPD-64PP (simulations S14 and S20) or form a closed gap in the case of XPD-CPD (S13). The initial structures for each model are the first snapshot after minimization in the MD simulations.

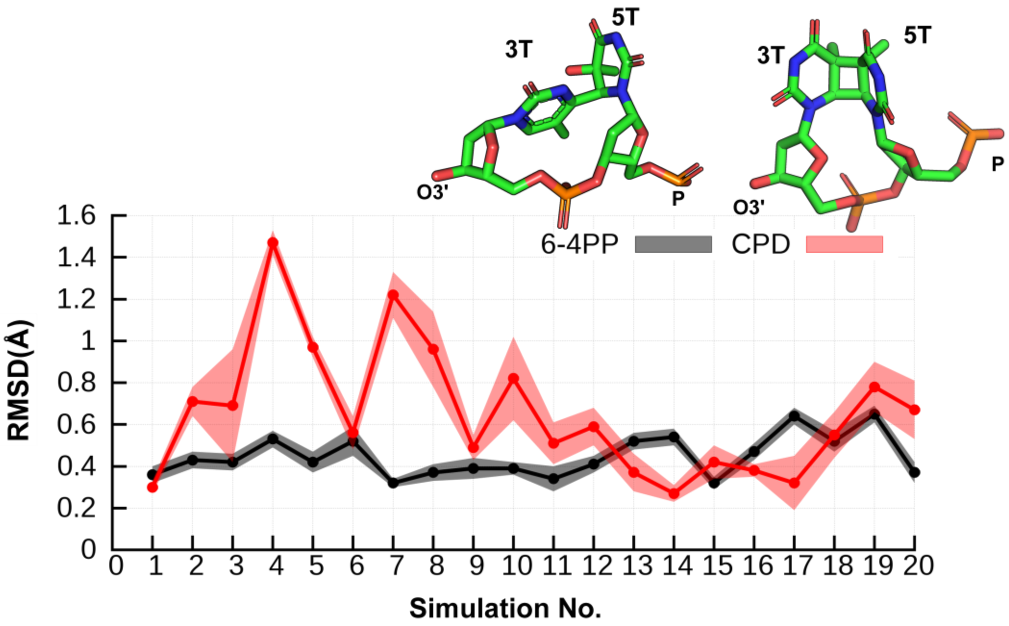


**Supplementary Figure S4.** The 6−4PP lesion is more rigid than CPD regardless of its positioning near the entry pore.

The RMSD plot shows the motion of the 3T base relative to the 5T base in each lesion. The ensemble average values and the standard deviations of the heavy atom RMSDs of the 3T base are obtained after fitting the 5T heavy atoms to the initial structure for each simulation. The ensemble average values are shown as dots connected by a solid line and the standard deviations are highlighted in shadow.

The 6−4PP dimer is rigid, characterized by the small, similar standard deviations of the RMSDs. Due to its rigidity, its nearly perpendicular dimers are similar to their initial structures with average RMSDs less than 0.64 Å. In CPD, the 3T base deviates from its initial structure by up to ~ 1.5 Å in RMSD (ranging from 0.2 to 1.5 Å), indicating CPD dimers can adopt conformations different from their initial state. The CPD cyclobutane ring is flexible and allows conformational mobility.

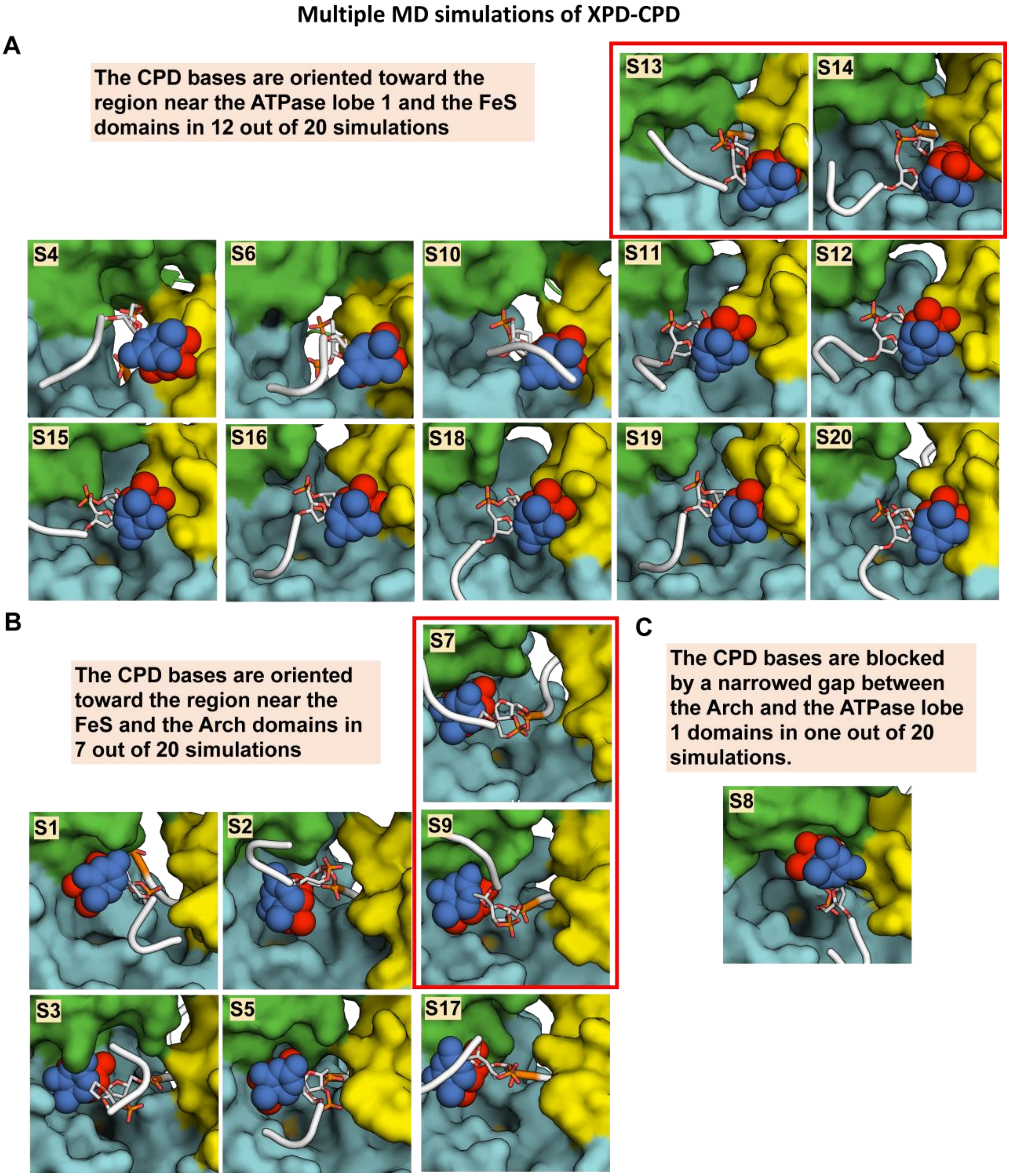


**Supplementary Figure S5.** CPD is completely blocked from entering into the pore with varying orientations outside the XPD entrance.

The 20 independent MD simulations revealed that CPD adopts varying orientations outside the pore entry and its bases are completely blocked from entering the pore. The CPD dimer displays three main orientations outside the entry pore, as shown in (A)−(C). Full details concerning the

XPD-lesion vdW interactions corresponding to each simulation are given in **Supplementary Tables S1B and S2B**.

(**A**) 12 of 20 simulations (*i.e.*, 60 % of the population) showed the bases oriented toward the region near the ATPase lobe 1 and the FeS domains. All these cases revealed that the bases form vdW interactions mainly with the ATPase lobe 1 domain. The strongest vdW interactions of the bases with XPD are observed in the cases (S13 and S14) where the modified bases that interact with the ATPase lobe 1 domain also form the greatest vdW interactions with the FeS domain (**Supplementary Tables S2B**).

(**B**) In 7 of the 20 simulations (*i.e.*, 35% of the population), the modified bases are oriented toward the region near the FeS and the Arch domains. All these cases revealed that the bases form vdW interactions mainly with the FeS domain. Strong vdW interactions of the bases with XPD are found in the cases (S7 and S9) where the vdW interactions with the FeS and Arch domains are both the greatest (**Supplementary Tables S1B**).

(**C**) In 1 of the 20 simulations (*i.e.*, 5 % of the population), the modified bases are pointed away from the FeS domain and toward the gap between the Arch and the ATPase lobe 1 domain, concomitant with a narrowed gap between these two domains.

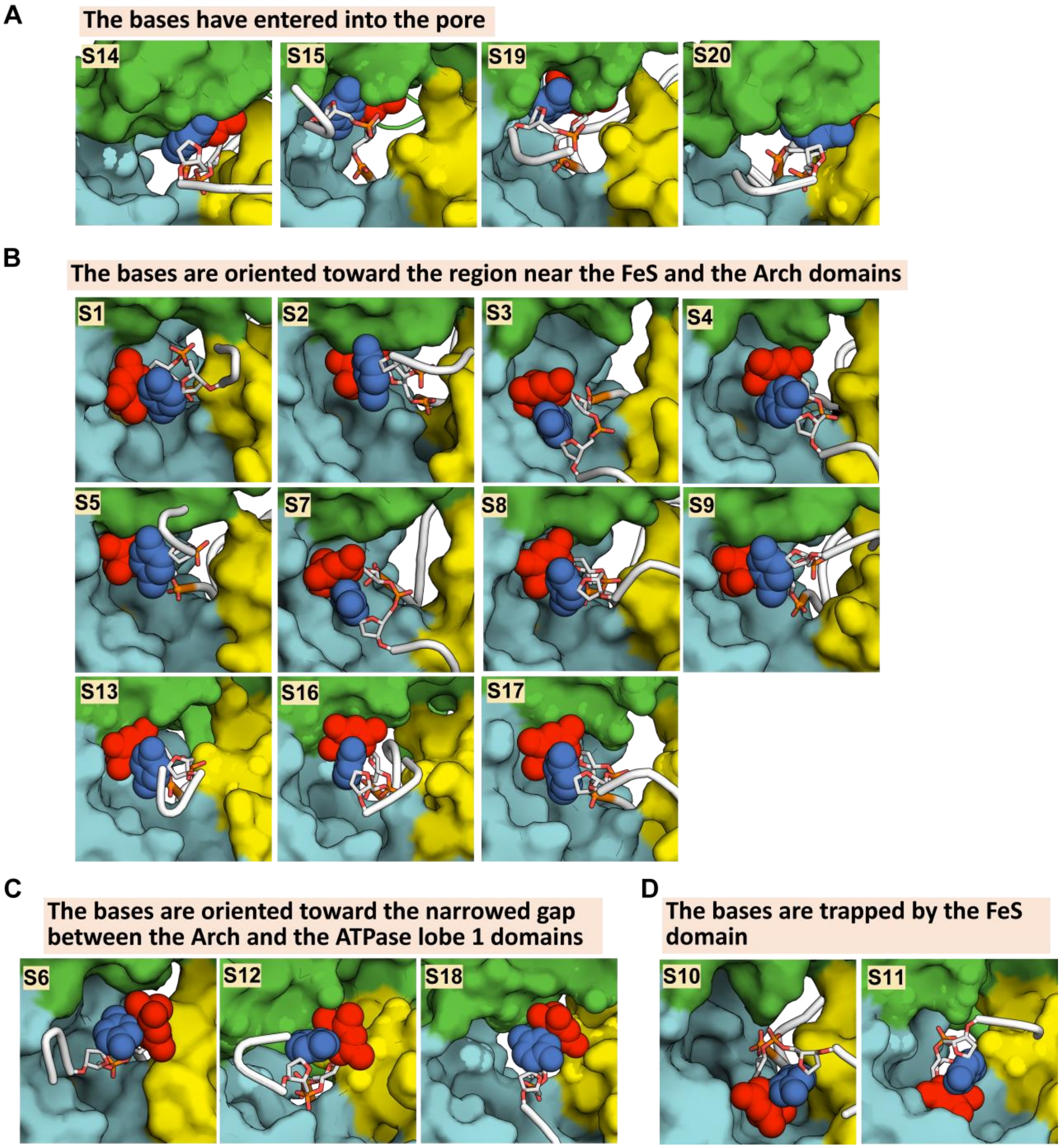


**Supplementary Figure S6**. Multiple MD simulations reveal that the modified bases of 6−4PP can be translocated into the pore. Blockage of the bases occurs mainly when they are oriented toward the region near the Arch and the FeS domains.

(**A**) In 4 out 20 simulations (*i.e.*, 20 % of the population), 6−4PP initially positioned outside the pore is flipped into the unoccupied space within the pore via translocation of its modified bases. In these cases, the Vdw interactions of the bases with the Arch domain are the greatest (**Supplementary Tables 4B and 5B**).

(**B−D**) Sixteen of 20 simulations (*i.e.*, 80 % of the population) revealed blockage of the 6−4PP lesion from entry into the pore. Among these, (**B**) 11 show that the bases of 6−4PP are oriented

toward the interface between the Arch and the FeS domains; (**C**) 3 reveal that the bases are positioned at the gap between the Arch and the ATPase lobe 1 domains, and (**D**) two simulations showed the bases trapped by the region of the FeS domain. Full details concerning the XPD-lesion interactions corresponding to each simulation are given in **Supplementary Tables S4-S6**.

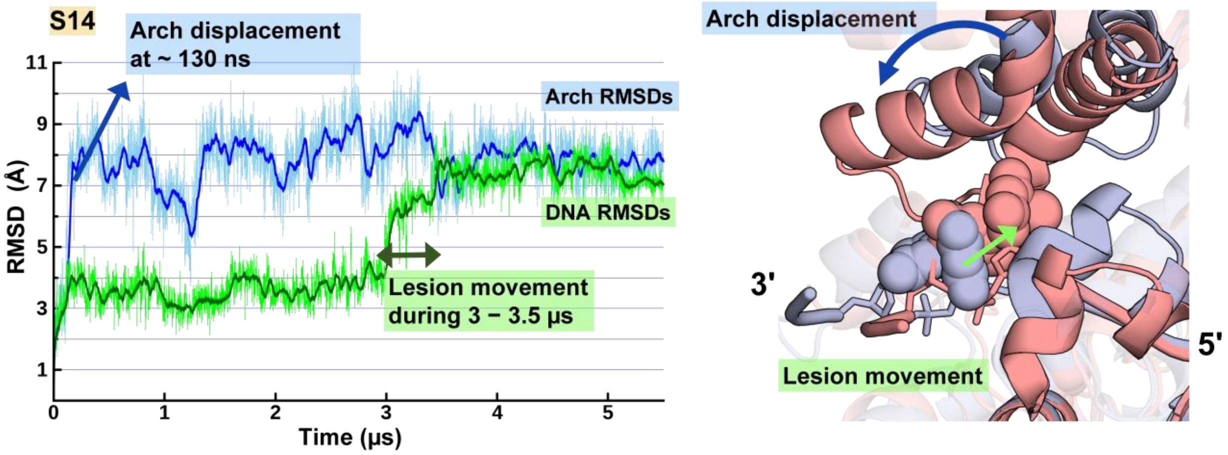


**Supplementary Figure S7**. Simulation S14 of XPD-64PP shows that the base-translocation of 6−4PP occurs during 0 ~ 3.5 μs via two conformational transitions.

Superimposed structures with 6−4PP prior to translocation (light-blue) and after translocation (light-red) with a view along the entry pore show that two main movements, taking place at different times, contribute to the translocation of 6−4PP's bases into the pore. One is the shift of the Arch domain (dark-blue arrow) and the other is the movement of the lesion in a 3' to 5' direction (green arrow). For the movement of the Arch domain, we computed the relative motion of the Arch and the FeS domains near the entry pore by monitoring the Cα RMSD (blue line) of the Arch domain after fitting the stable region of the FeS Cα atoms to the initial structure. At ~ 130 ns, the Arch domain deviates significantly from its initial structure by more than ~ 8 Å. For the movement of the lesion, we computed the RMSDs of heavy-atoms (green line) of the lesion while superimposing the XPD pore. The 3' to 5' diretional movement of 6−4PP occurs during the transition between ~ 3 and ~ 3.5 μs. In the plot of time-dependent RMSDs, only the first 5.5 μs was shown to display the pathway for the translocation of the lesion via its bases entering into the pore, which takes place during 0 ~ 3.5 μs via two conformational transitions.

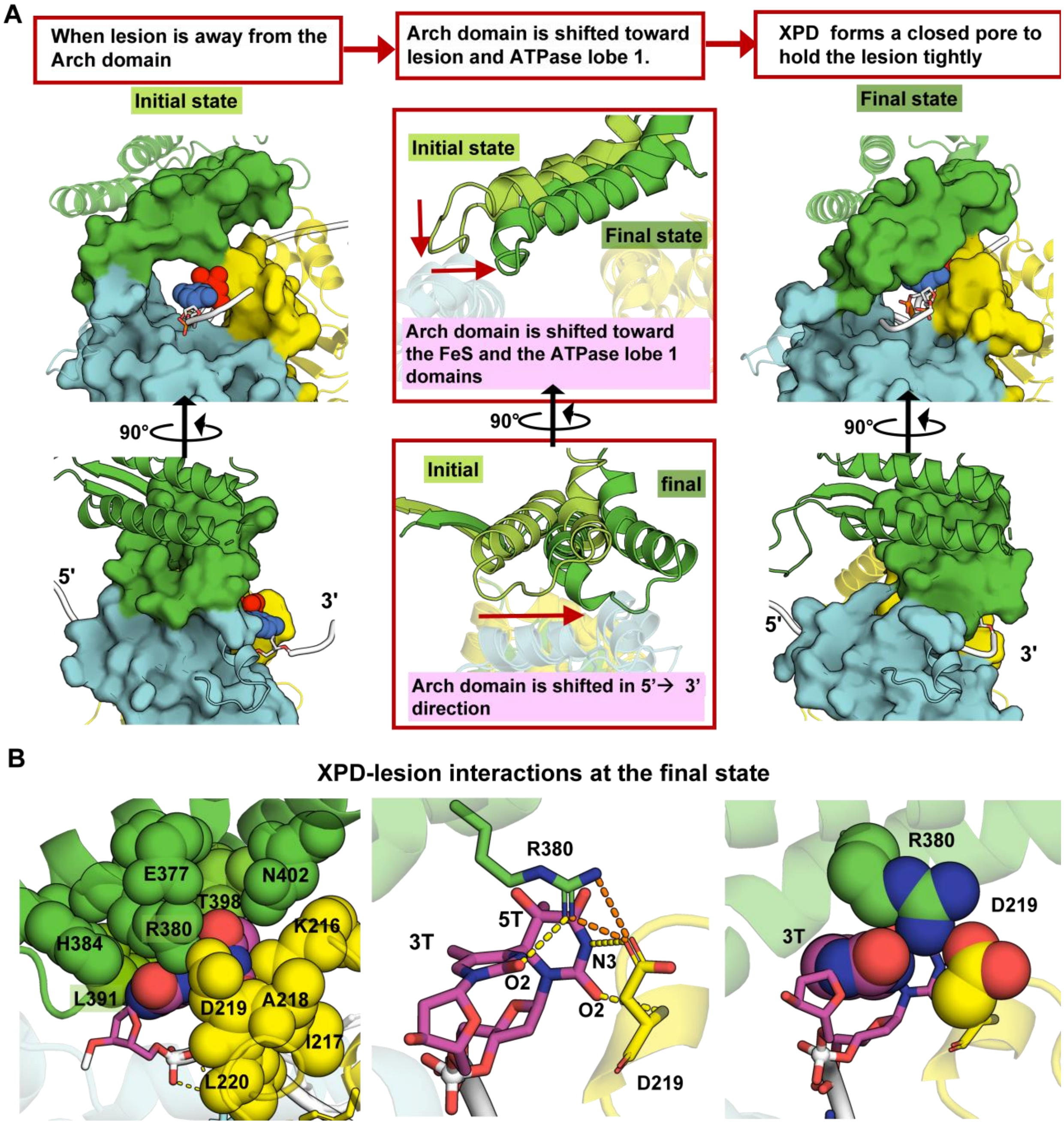


**Supplementary Figure S8**. Simulation S20 of XPD-64PP shows the greatest vdW interactions of the XPD pore with the 6−4PP lesion when the lesion is translocated into the pore.

(**A**) When the lesion is initially oriented toward the ATPase lobe 1 domain, the Arch domain is shifted toward the ATPase lobe 1 and the FeS domains as well as the 3'-DNA. As a result, the 6−4PP lesion is tightly bound between the Arch and the ATPase lobe 1 domain.

(**B**) The extensive interactions of the XPD pore and the bases of 6−4PP within the pore.

**Left panel**: the modified bases are clamped tightly between the Arch and the ATPase lobe 1 domains. The side-chains of the Arch residues R380, H384, L391, T398, N402, and the ATPase

lobe 1 helix residues 216 − 220 form stable van der Waals interactions with the 5T and 3T bases of the 6−4PP. The N3 and O2 atoms of the 5T base are captured by a small pocket formed by the ATPase lobe 1 helix. The O4 atom and methyl group of 5T are held between R380, T398, and N402 of the Arch domain.

**Middle panel**: the modified bases form two hydrogen bonds with the residues R380 and D219 (details see S20 in **Supplementary Table S6**). In the 5T base of 6−4PP, the O2 atom of 5T is hydrogen-bonded to the N atom of D219 and the 5T N3 atom is hydrogen-bonded to the OD1 and OD2 atoms of D219; in the 3T base, the N3 and O2 atoms are hydrogen-bonded to the R380 Nη1 atoms (yellow dashed-lines). Furthermore, the Nη1 atoms of R380 are hydrogen-bonded to the OD1 and OD2 atoms of D219 (orange dashed-lines), contributing to the stabilization of the 6−4PP lesion within the pore.

**Right panel**: R380 is accommodated neatly with its side-chain stacking with the 5T base and it is also hydrogen-bonded with the 3T base due to the nearly perpendicular, rigid bases of 6−4PP. Also, it’s side-chain crosses over the 3T base to point toward D219 of the ATPase lobe 1 domain, resulting in a very closed XPD pore.

**Supplementary Movies**

**Supplementary MovieS1.mp4**: Simulation (0 – 2.0 μs) of **XPD-CPD** reveals that CPD initially positioned outside the DNA entry pore undergoes a backbone-translocation into the pore, but its bases are blocked from entering.

**Supplementary MovieS2.mp4**: Simulation (0 – 5.3 μs) of **XPD-64PP** reveals that 6−4PP initially positioned outside the DNA entry pore is translocated in the 3' to 5' direction via its bases flipping into the unoccupied space within the pore.

**Supplementary References**

[1] J.A. Maier, C. Martinez, K. Kasavajhala, L. Wickstrom, K.E. Hauser, C. Simmerling, ff14SB: Improving the cccuracy of protein side chain and backbone parameters from ff99SB, J. Chem.Theory Comput. 11 (2015) 3696-3713.

[2] D. Paul, H. Mu, H. Zhao, O. Ouerfelli, P.D. Jeffrey, S. Broyde, J.H. Min, Structure and mechanism of pyrimidine-pyrimidone (6-4) photoproduct recognition by the Rad4/XPC nucleotide excision repair complex, Nucleic Acids Res 47 (2019) 6015-6028.

[3] H. Mu, N.E. Geacintov, Y. Zhang, S. Broyde, Recognition of Damaged DNA for Nucleotide Excision Repair: A Correlated Motion Mechanism with a Mismatched cis-syn Thymine Dimer Lesion, Biochemistry 54 (2015) 5263-5267.

[4] A.T.P. Carvalho, M. Swart, Electronic Structure Investigation and Parametrization of Biologically Relevant Iron–Sulfur Clusters, Journal of Chemical Information and Modeling 54 (2014) 613-620.

[5] W.L. Jorgensen, J. Chandreskhar, J.D. Madura, R.W. Imprey, M.L. Klein, Comparison of simple potential functions for simulating liquid water, J. Chem. Phys. 79 (1983) 926-935.

[6] D.A. Case, Ben-Shalom, I.Y., Brozell, S.R., Cerutti, D.S., Cheatham, T.E. III, Cruzeiro, W.D. V., Darden, T.A., Duke, R.E., Gilson, M.K., Gohlke, H., Goetz, A.W., Greene, D., Harris, R., Homeyer, N., Huang, Y., Izadi, S., Kovalenko, A., Kurtzman T, Lee, T.S., LeGrand, S., Li, P., Lin, C., Liu, J., Luchko, T., Luo, R., Mermelstein, D.J., Merz,. K.M., Miao, Y., Monard, G., Nguyen, C., Nguyen, H., Omelyan, I., Onufriev, A., Pan, F., Qi, R., Roe, R. D., Roitberg, A., Sagui, C., Schott-Verdugo, S., Shen, J., Simmerling, C.L., Smith, J., SalomonFerrer, R., Swails, J., Walker, R.C., Wang, J., Wei, H., Wol,f R.M., Wu, X., Xiao, L., York, D.M. and Kollman, P.A., AMBER2018, University of California, San Francisco, 2018.

[7] I. Fu, H. Mu, N.E. Geacintov, S. Broyde, Mechanism of lesion verification by the human XPD helicase in nucleotide excision repair, Nucleic Acids Res 50 (2022) 6837-6853.

[8] T. Darden, D. York, L. Pedersen, Particle mesh Ewald: an $N \log(N)$ method for Ewald sums in large systems, J. Chem. Phys. 98 (1993) 10089-10092.

[9] T.E. Cheatham, J.L. Miller, T. Fox, T.A. Darden, P.A. Kollman, Molecular-Dynamics simulations on solvated biomolecular systems - the particle mesh Ewald eethod leads to stable trajectories of DNA, RNA, and proteins, J. Am. Chem. Soc. 117 (1995) 4193-4194.

[10] D.R. Roe, T.E. Cheatham, PTRAJ and CPPTRAJ: Software for processing and analysis of molecular dynamics trajectory Data, J. Chem. Theory Comput. 9 (2013) 3084-3095.

[11] H. Flyvbjerg, H.G. Petersen, Error estimates on averages of correlated data, The Journal of Chemical Physics 91 (1989) 461-466.

[12] W. Yang, R. Bitetti-Putzer, M. Karplus, Free energy simulations: use of reverse cumulative averaging to determine the equilibrated region and the time required for convergence, J Chem Phys 120 (2004) 2618-2628.

[13] Schrodinger, LLC, The PyMOL Molecular Graphics System, Version 1.8, in, 2015.

[14] W. Humphrey, Dalke, A., and Schulten, K., VMD - Visual Molecular Dynamics., Journal of Molecular Graphics 14 (1996) 33-38.

[15] G. Kokic, A. Chernev, D. Tegunov, C. Dienemann, H. Urlaub, P. Cramer, Structural basis of TFIIH activation for nucleotide excision repair, Nat Commun 10 (2019) 2885.

[16] J.Y. Shao, S.W. Tanner, N. Thompson, T.E. Cheatham, Clustering molecular dynamics trajectories: 1. Characterizing the performance of different clustering algorithms, J. Chem. Theory Comput. 3 (2007) 2312-2334.
